# Have the cake and eat it: Optimizing nondestructive DNA metabarcoding of macroinvertebrate samples for freshwater biomonitoring

**DOI:** 10.1111/1755-0998.13012

**Published:** 2019-04-29

**Authors:** Filipa M. S. Martins, Mafalda Galhardo, Ana F. Filipe, Amílcar Teixeira, Paulo Pinheiro, Joana Paupério, Paulo C. Alves, Pedro Beja

**Affiliations:** ^1^ Departamento de Biologia Faculdade de Ciências da Universidade do Porto Porto Portugal; ^2^ CIBIO/InBio, Centro de Investigação em Biodiversidade e Recursos Genéticos Universidade do Porto Vairão Portugal; ^3^ CIBIO/InBio, Centro de Investigação em Biodiversidade e Recursos Genéticos, Instituto Superior de Agronomia Universidade de Lisboa Lisboa Portugal; ^4^ CIMO‐ESA‐IPB, Centro de Investigação de Montanha Instituto Politécnico de Bragança Bragança Portugal; ^5^ AQUALOGUS ‐ Engenharia e Ambiente, Lda. Lisboa Portugal; ^6^ Wildlife Biology Program University of Montana Missoula Montana

**Keywords:** benthic macroinvertebrates, DNA extraction, DNA metabarcoding, freshwater bioassessment, preservative ethanol, Water Framework Directive

## Abstract

DNA metabarcoding can contribute to improving cost‐effectiveness and accuracy of biological assessments of aquatic ecosystems, but significant optimization and standardization efforts are still required to mainstream its application into biomonitoring programmes. In assessments based on freshwater macroinvertebrates, a key challenge is that DNA is often extracted from cleaned, sorted and homogenized bulk samples, which is time‐consuming and may be incompatible with sample preservation requirements of regulatory agencies. Here, we optimize and evaluate metabarcoding procedures based on DNA recovered from 96% ethanol used to preserve field samples and thus including potential PCR inhibitors and nontarget organisms. We sampled macroinvertebrates at five sites and subsampled the preservative ethanol at 1 to 14 days thereafter. DNA was extracted using column‐based enzymatic (TISSUE) or mechanic (SOIL) protocols, or with a new magnetic‐based enzymatic protocol (BEAD), and a 313‐bp COI fragment was amplified. Metabarcoding detected at least 200 macroinvertebrate taxa, including most taxa detected through morphology and for which there was a reference barcode. Better results were obtained with BEAD than SOIL or TISSUE, and with subsamples taken 7–14 than 1–7 days after sampling, in terms of DNA concentration and integrity, taxa diversity and matching between metabarcoding and morphology. Most variation in community composition was explained by differences among sites, with small but significant contributions of subsampling day and extraction method, and negligible contributions of extraction and PCR replication. Our methods enhance reliability of preservative ethanol as a potential source of DNA for macroinvertebrate metabarcoding, with a strong potential application in freshwater biomonitoring.

## 1 INTRODUCTION

Freshwater ecosystems are among the most threatened ecosystems in the world, facing numerous pressures associated with pollution, eutrophication, damming and regulation of rivers, water overuse, invasive species and climate change (Craig et al., 2017; Vörösmarty et al., 2010). These drivers are leading to fast biodiversity declines and hindering services provided by freshwater ecosystems (Craig et al., 2017; Vörösmarty et al., 2010). To counteract these trends, national and international regulations have been enacted to protect and rehabilitate freshwater ecosystems, such as the Water Framework Directive (WFD, Directive 2000/60/EC) applied in the European Union. These regulations involve country‐specific, long‐term and large‐scale monitoring programmes, requiring the development of cost‐effective methodologies to assess the ecological status of aquatic ecosystems (Birk et al., 2012; Pawlowski et al., 2018).

Currently, freshwater biological assessments around the globe are generally based on the characterization of communities of indicator organisms, which are used to derive biotic indices quantifying the biological quality status (Birk et al., 2012; Pawlowski et al., 2018). For example, assessments in rivers under the WFD include indicator organisms as diatoms, macroalgae and angiosperms, benthic invertebrates and fish (Birk et al., 2012). Typically, the monitoring programmes involve sampling at field sites, sample preparation (e.g. sorting), morphological species identification and quantification, calculation of biotic indices and quality assessment (Pawlowski et al., 2018). Although this approach has been successfully used since the mid‐20th century, it is labour‐intensive and time‐consuming, which in many cases may limit the number of sites that can be sampled, and the frequency of sampling (Hajibabaei, Shokralla, Zhou, Singer, & Baird, 2011). The need for morphological identification of organism is particularly troublesome, as this is laborious and requires taxonomic expertise that is often very limited. Also, for many organisms, misidentifications may occur or identifications may be impossible to achieve at the highest taxonomical resolution required for fine ecological assessments, due to difficulties in identifying certain groups and/or life stages (e.g. larvae of some macroinvertebrates) (Hajibabaei et al., 2011). Given these difficulties and the advent of powerful high‐throughput DNA sequencing, there has been an increasing interest in the use of molecular tools in ecosystem assessment (Sweeney, Battle, Jackson, & Dapkey, 2011; Taberlet, Coissac, Pompanon, Brochmann, & Willerslev, 2012), now often referred as biomonitoring 2.0 (Baird & Hajibabaei, 2012).

DNA metabarcoding may be particularly useful in freshwater biomonitoring because it is able to process complex multispecies assemblages, and is potentially faster, lower‐priced and more refined than conventional methods (Aylagas, Borja, Irigoien, & Rodríguez‐Ezpeleta, 2016; Gibson et al., 2014; Hajibabaei et al., 2011). By combining DNA taxonomic identification, high‐throughput sequencing and bioinformatic pipelines, metabarcoding can achieve higher taxonomic resolution and thus potentially higher sensitivity of metrics to fine variations in freshwater ecosystems (Andújar et al., 2018; Carew, Pettigrove, Metzeling, & Hoffmann, 2013; Gibson et al., 2015). Despite its potential, there are still several technical and conceptual challenges associated with the use of DNA metabarcoding in freshwater bioassessment (Leese et al., 2016; detailed revision in Pawlowski et al., 2018), which need to be addressed before it can be mainstreamed into official monitoring programmes such as those undertaken under the WFD (Leese et al., 2016; Pawlowski et al., 2018). In the case of biotic indices based on benthic macroinvertebrates, for instance, one of the problems is the need for pre‐processing bulk samples, such as cleaning and sorting of specimens before DNA extraction (Aylagas et al., 2016; Elbrecht, Peinert, & Leese, 2017; Elbrecht, Vamos, Meissner, Aroviita, & Leese, 2017), which increase processing time, costs and possible contamination. Furthermore, DNA extraction from a bulk sample requires its destruction, which may be problematic if the sample is required for other uses, or if it needs to be preserved for a certain period of time due to legal reasons, as currently required by some regulatory agencies.

In this study, we optimize and evaluate procedures for nondestructive DNA metabarcoding of macroinvertebrate samples used for freshwater bioassessment. Our approach is based on DNA extraction from the ethanol used to preserve macroinvertebrate bulk samples in the field without preprocessing, and thus including plant materials, detritus, stones and nontarget organisms. Previous studies have already shown the possibility of obtaining macroinvertebrate DNA from ethanol used to preserve clean and sorted bulk samples, or single specimens (Hajibabaei, Spall, Shokralla, & Konynenburg, 2012; Linard, Arribas, Andújar, Crampton‐Platt, & Vogler, 2016; Shokralla, Singer, & Hajibabaei, 2010). So far, only Zizka, Leese, Peinert, and Geiger (2018) have dealt with “dirty” ethanol, which includes a wider array of contaminants and potential PCR inhibitors, comparing the performance of different treatments prior to DNA extraction to increase DNA concentration in the preservative ethanol. Here, we aimed to evaluate how the efficiency in recovering macroinvertebrate taxa using metabarcoding was affected by the timing of ethanol subsampling (1 to 14 days after field sampling) and DNA extraction methods, and to further demonstrate the potential use of DNA extracted from “dirty” preservative ethanol to identify macroinvertebrate taxa from coarse bulk samples when compared to traditional methods. In addition, we assessed the consistency of metabarcoding results across extraction and PCR replicates. Our results were used to discuss the potential application of ethanol‐based approaches in the biological monitoring of freshwaters using macroinvertebrate indicators.

## 2 MATERIAL AND METHODS

### 2.1 Field sampling

Macroinvertebrates were collected from five stream reaches within the Tua River watershed (NE Portugal) in 2015 (Figure S1), following the standardized sampling methodology established officially in Portugal under the WFD (Instituto da Água (INAG) 2008). Briefly, at each sampling site a 50‐m sector of stream was selected, and six macroinvertebrate subsamples were collected by kick‐sampling using a dip net with 0.25‐m opening and 500 µm mesh size, covering proportionally the most representative habitats. Each subsample involved kick/sweep sampling of 1 m stream length in the upstream direction. All six subsamples within a site were pooled into a single bulk sample, preserved in ethanol 96% with an approximate ethanol:bulk ratio of 3:1 and stored at room temperature until the end of the experiment. Ethanol concentration was similar to that used by Shokralla et al. (2010) and Zizka et al. (2018), and it was expected to be more effective at preserving DNA and bulk samples than the concentration of 70% used in other studies (Elbrecht, Vamos, et al., 2017; Stein, White, Mazor, Miller, & Pilgrim, 2013). Linard et al. (2016) used 100% ethanol, but this is far more costly and may be less amenable for large‐scale field surveys. We used a constant ethanol:bulk ratio instead of a constant volume of ethanol because the later might lead to variations in DNA concentration in the preservative solution inversely related to the amount of biological material collected in the field, and could thus reduce comparability of results across sampling sites.

### 2.2 Laboratory procedures

After careful manual shaking, five 2‐ml subsamples of preservative ethanol were taken from each macroinvertebrate bulk sample on days 1, 2, 3, 5, 7 and 14 following field sampling and stored at −20ºC until DNA extraction. The subsample volume was chosen as a balance between the objective of representing macroinvertebrate diversity in the bulk sample, and the need to take many replicate subsamples from each bulk throughout the experiment. The duration of the experiment was established based on the range used in other studies (e.g. Linard et al., 2016; Zizka et al., 2018), and considering a prior expectation that results would stabilize in about two weeks. Furthermore, a relatively short period was tested because National Regulatory Authorities need to have water quality information as soon as possible upon field sampling. A higher frequency of subsampling was carried out in the first days because this was the period when we expected the results to change more rapidly.

Prior to DNA extraction, ethanol was completely evaporated using an Eppendorf vacuum concentrator. Genomic DNA was then extracted from each 2‐ml preservative ethanol subsample using one out of three extraction methods (Table S1; Supplementry Methods): [SOIL], NucleoSpin® Soil (MACHEREY‐NAGEL GmbH & Co, Düren, Germany); [TISSUE], a modified E.Z.N.A.® Tissue DNA Kit protocol (Omega Bio‐tek, Inc., Georgia, United States) with InhibitEX® Buffer (QIAGEN, Hilden, Germany); and [BEAD], a newly developed protocol using Agencourt AMPure XP® beads (A Beckman Coulter Company, Massachusetts, United States) and Qiagen® buffers. TISSUE was used in three subsamples per site/day to check for consistency across extraction replicates, while SOIL and BEAD were used in one subsample per site/day each. We selected TISSUE to test for consistency because preliminary testing indicated that this was the best performing of the two commercial kits considered in our study (SOIL and TISSUE). The extracted DNA was eluted in 70 μl and then diluted one time with ultrapure water prior to amplification to increase PCR amplification success. Extraction negative controls containing all reagents except the ethanol subsample were included. The quantity (ng/μl) and integrity of extracted DNA were assessed using an Agilent 2200 TapeStation system (Agilent Technologies, Inc., California, USA). DNA integrity was evaluated using the DIN (DNA Integrity Number) algorithm estimated with Genomic DNA ScreenTape, which is based on the size distribution of DNA fragments and varies between 1 (highly degraded) and 10 (highly intact).

Library preparation was performed in two steps, adapted from the protocol described by Kircher, Sawyer, and Meyer (2011) and Gansauge and Meyer (2013). First‐round PCR amplifications were performed using the reverse primer BR2 (Elbrecht & Leese, 2017) and a redesigned forward primer (MARTINS‐2019‐COI_Fw, 5´‐GGNTGAACHGTHTAYCCHCC‐3´) from the Ill_C_R (Shokralla et al., 2015) reversed complemented. These primers were used because preliminary in vitro testing showed their ability to consistently amplify Ephemeroptera, Plecoptera, Trichoptera and Odonata (EPTO) taxa, which are widely considered the best macroinvertebrate indicators of freshwater biological quality (Bonada, Prat, Resh, & Statzner, 2006), and for which we had a considerable barcode database from specimens collected within or close to the study area (IBI, CIBIO‐InBIO Barcoding Initiative). Each 10‐μl PCR mixture contained 5 μl of hotstart master mix (Multiplex PCR Kit, QIAGEN), 0.4 μl of each primer (10 μM stock), 2.2 μl ultrapure water and 2 μl of diluted DNA. Initial tests failed when using the 2x KAPA HiFi HotStart ReadyMix (Kapa Biosystems, Cape Town, South Africa) recommended for amplicon library preparation by Illumina. The Qiagen polymerase improved amplification success in ethanol samples, as observed by Nichols et al. (2018). After an initial denaturation cycle at 95°C for 15 min, 38 cycles of 30 s at 95°C, 60 s annealing at 50°C and 30 s extension at 72°C were performed, followed by a final elongation at 60°C for 10 min. Each sample, including extraction negative controls, was replicated three times, and PCR negative controls containing no template DNA were also included. PCR amplicons were then visualized on a 2% agarose gel and diluted ten times prior to indexing PCR (second‐round PCR). A few samples showed weak bands and were not diluted.

Unique dual indexes were selected for each sample and each 10‐μl indexing PCR mixture contained 5 μl 2x KAPA HiFi HotStart ReadyMix (Kapa Biosystems, Cape Town, South Africa), 1 μl of mixed indexing primer (5 μM stock; Gansauge & Meyer, 2013), 2 μl ultrapure water and 2 μl of diluted first‐round PCR product. Indexing thermal cycling conditions were 95°C, for 3 min; followed by 10 cycles of 95°C for 30 s, 55°C for 30 s, 72°C for 30 s, with an extension of 72°C for 5 min. A different annealing temperature was used in indexing PCR to guarantee library quality. Indexing PCR success was evaluated through electrophoresis in 15% of the samples, and then, final sample libraries were purified using 1.2x AMPure®XP beads. Each sample library was quantified by fluorometry using Quant‐iT™ PicoGreen®dsDNA Assay Kit (Life Technologies, California, USA) and normalized before pooling. The final library was then validated in the TapeStation system (High Sensitivity D1000 ScreenTape Assay) and normalized to 4 nM after quantification in qPCR using KAPA Library Quantification Kit for Illumina platforms. Dual‐indexed PCR amplicons were sequenced in an Illumina MiSeq System using one MiSeq V2 500‐cycle reagent kit (Illumina, California, USA) with paired‐end reads.

At the end of the experiment, the bulk samples were cleaned and sorted, and the WFD‐targeted macroinvertebrate taxa were morphologically identified to the lowest possible taxonomic level. A particular effort was taken to achieve species‐level identification for EPTO taxa, since many had been identified at this level from metabarcoding data.

### 2.3 Bioinformatic analysis

Sequence reads were processed using the OBITools software suite (Boyer et al., 2016), from pairwise alignment to clustering (Bálint, Márton, Schatz, Düring, & Grossart, 2018; Taberlet, Bonin, Zinger, & Coissac, 2018), following procedures detailed in the Supplementry Methods (see Supporting Information). Particular care was taken to remove artefacts resulting from PCR and sequencing errors, including the use of “obigrep” to eliminate sequences with a length outside the expected metabarcode size (310–316), and sequences occurring just once across the data set. The command “obiclean” was used to filter out potentially erroneous sequences compatible with indel or substitution errors, based on their lower frequency of occurrence and similarity to most common sequences (De Barba et al., 2014). We also removed resulting cluster sequences with ≤0.03% of read coverage in at least one sample and ≤5 reads. Finally, extraction and PCR negative controls were used to filter out potential contaminants.

Each cluster sequence was taxonomically assigned considering three databases: using BLAST searches against NCBI Nucleotide database “nt” (downloaded in September 2017) and our private species database (IBI—InBIO Barcoding Initiative); and using the search engine of the BOLD database (details in Supplementry Methods in Supporting Information). Assignments to species level were required to have a percentage identity of at least 98%, whereas lower identity thresholds were required for assignments to Order or lower taxonomic levels (<92% of identity), Family (≥92%) and Genus (≥95%) levels. Assignments were cross‐checked using the three databases, and the best match was retained. All assignments were manually checked for plausibility, including verification of the likelihood of species occurrence close to the study area, using information on species geographic range and occurrence records.

In the case of macroinvertebrate taxa targeted by the WFD in Portugal (i.e. species with aquatic life stages of the Orders Turbellaria, Gastropoda, Bivalvia, Oligochaeta, Hirudinea, Crustacea and Insecta; INAG, 2008), a tree‐based approach was used to classify the cluster sequences not assigned to species as phylogenetic divergent units (phylOTU; Sharpton et al., 2011). For this, sequences assigned to the same Order were aligned and clustered hierarchically using Unweighted Pair Group Method with Arithmetic Mean (UPGMA) trees based on HKY distance model (1,000 bootstrap replicates), in geneious v10 (Kearse et al., 2012). This approach was used to visually detect spurious sequences that might have passed through the pipeline filtering (including pseudogenes), and define group‐specific divergence thresholds. Assuming distance thresholds derived from sequence databases, we considered a distinct phylOTU each cluster of sequences that was separated from all other clusters by ≥5%, except in the case of the Trichoptera and Hemiptera for which we selected a threshold of 3% (further details in Supplementry Methods in Supporting Information). Species and phylOTU data were combined in a single matrix to analyse the diversity and composition of WFD and EPTO taxa communities. Because rare occurrences can result for instance from cross‐contamination or tag jumps during the process (Taberlet et al., 2018), species/phylOTU with a read coverage <0.01% were removed from each sample. As the criteria and thresholds to remove rare taxa can influence results, analyses were repeated with the unfiltered taxa matrix, with the exclusion of “singleton” taxa from the matrix (i.e. taxa with only one read) and with the matrix trimmed at 0.03% and 0.05% thresholds. Results are presented considering the 0.01% threshold except where indicated otherwise.

### 2.4 Statistical analysis

Our study was based on a hierarchically structured design that considered five stages: sampling site (*n* = 5), subsampling day (*n* = 6), extraction method (*n* = 3), extraction replicate (*n* = 1 in SOIL and BEAD; or *n* = 3 in TISSUE) and PCR replicate (*n* = 3). The first three stages are crossed, and the last two are nested within the hierarchical stages above (Schielzeth & Nakagawa, 2013). The experiment thus produced 450 sampling units, of which only 418 were carried out for subsequent analysis once units producing <500 reads of WFD‐targeted taxa were discarded. For each sampling unit, we estimated species richness as the total number of WFD taxa detected through metabarcoding. We also used Chao1 estimator of species richness, thereby accounting for differences on sampling effort and sample completeness (Chao & Chiu, 2016). The Chao1 estimator was only computed on the unfiltered taxa matrix, because estimation is based on the numbers of singletons and doubletons (Chao & Chiu, 2016), and thus should not be used with trimmed data (McMurdie & Holmes, 2013). Richness variables were calculated using the R phyloseq package (McMurdie & Holmes, 2013).

Generalized additive mixed models (GAMM) were used to model variation in each response variable in relation to independent variables and their interactions, which permit detecting nonlinear responses without needing a priori assumptions on the expected shape of such responses, while accounting for the hierarchical structure of the experiment (Wood, 2006). In the fixed component of the GAMM, we considered as independent variables the extraction method, the subsampling day and the interaction between the two. The fixed component also included the number of reads of the target taxa (i.e. WFD or EPTO taxa), thereby accounting for variation introduced by differences in coverage between samples. This approach was used instead of computing a rarefaction curve and truncating data considering a given read count threshold (Taberlet et al., 2018), because explicitly modelling the effects of coverage is increasingly considered a more robust and statistically efficient approach (McMurdie & Holmes, 2014). In GAMMs using either DNA concentration or integrity (DIN) as response variables, the nested random factors included the extraction replicate within extraction method within site. In GAMMs using either species richness or Chao1 diversity as response variables, the random component included the PCR replicate as an additional level nested within the other nested random factors. All GAMMs were built considering Gaussian errors and an identity link, except for observed species richness for which we used Poisson errors and a log link. In all models, we specified a penalized spline smoother with a basis dimension *k* = 4 for the subsampling day. The number of reads was log‐transformed, assuming a stabilization of the effects for high read counts. GAMMs were fitted using the package gamm4 (Wood & Scheipl, 2017) and plotted using the R ggplot2 package (Wickham, 2016).

To estimate the contributions of treatments and replicates to variation in community composition among units, we adopted the procedure of Mata et al. (2018), based on nonparametric permutational multivariate analysis of variance (PERMANOVA), using the adonis function (Oksanen et al., 2018). Specifically, we modelled the contribution of five components: (a) sites; (b) subsampling day within sites; (c) extraction method within subsampling day; (d) extraction replicate within extraction method; and (e) PCR replicate within extraction replicate. The contribution of each component while controlling for differences in degrees of freedom was estimated from the corresponding mean sum of squares (MSS). As a measure of the statistical significance of each component, we used an *F*‐statistic estimated with a permutation procedure (9,999 permutations). We used a hierarchical design because we were interested in estimating variation among subsampling days within each site, and not on variation among subsampling days per se. A similar reasoning applies to the other hierarchical levels. The read count (as log) was also included as an explanatory variable to account for variation in coverage among samples.

To estimate the percentage of matching between metabarcoding and morphological identification results for each sampling unit, we computed the proportion of taxa identified through morphological analysis that were retrieved through metabarcoding. As deviations between morphology and metabarcoding could also be due to taxa retrieved from the latter that were not detected by the former method, we computed Jaccard index as a measure of overall distance between each molecular sampling unit and the corresponding morphological sample. Separate comparisons were made for identifications at either the family or species level of EPTO, since identifications for most other taxa were often very coarse due to the lack of adequate barcode reference collections. To estimate how percentage matching and the Jaccard index varied in relation to extraction method and subsampling day, we used GAMMs with a fixed component and a nested random structure as described above for species richness. All analysis used morphological identification as the benchmark rather than metabarcoding the bulk sample itself, because we wanted to compare metabarcoding with the standard morphological approaches used in WFD monitoring programmes. In addition, as our bulk samples were collected under a WFD monitoring programme, they need to be preserved for at least five years and so could not be destroyed for bulk metabarcoding.

## 3 RESULTS

### 3.1 DNA concentration and integrity

**Figure 1:**
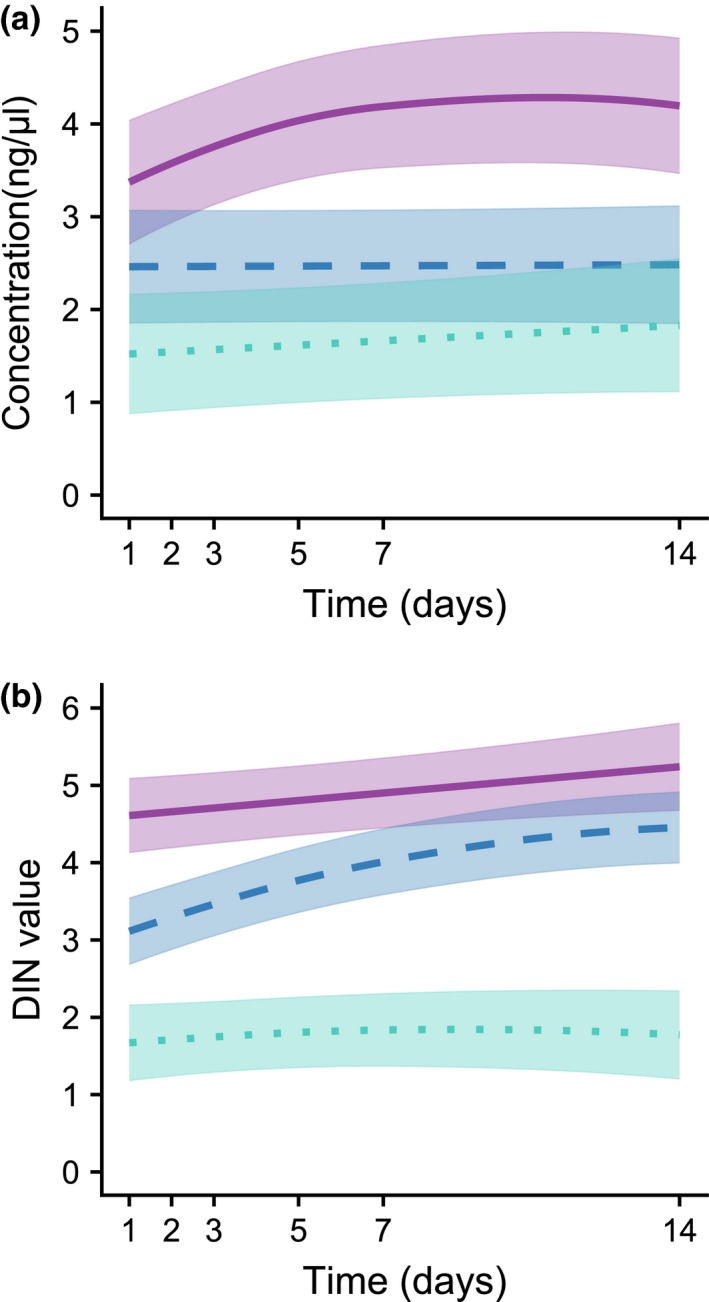
Variation predicted from GAMMs in (a) concentration (ng/μl) and (b) integrity given by Tapestation DNA Integrity Number (DIN) of DNA extracted from 96% ethanol used to preserve five unprocessed freshwater macroinvertebrate bulk samples, in relation to ethanol subsampling day and DNA extraction method. Subsampling was conducted at 1, 2, 3, 5, 7 and 14 days after field sampling, and DNA extractions were performed using three methods: BEAD (purple, solid line), TISSUE (blue, dashed line) and SOIL (green, dotted line). Temporal variation trend lines are provided with the corresponding standard errors. Summary statistics of the GAMMs are provided in Table 1 [Colour figure can be viewed at http://www.wileyonlinelibrary.com]

The concentration of DNA was significantly lower in samples extracted with SOIL than with BEAD extraction protocol (Figure 1a; Table 1). The subsampling day had no significant effects, though there was a tendency for increasing concentrations in samples extracted with BEAD up to about seven days. DNA integrity (DIN) was significantly lower in samples extracted with SOIL and TISSUE than with BEAD (Figure 1b; Table 1). There was a significant trend in DNA integrity increasing over time when using TISSUE, though at a slower rate after about the 7th day.

**Table 1:** Summary statistics of GAMM models relating DNA concentration (ng/μl) and DNA integrity given by Tapestation DNA Integrity Number (DIN) of DNA extracted from 96% ethanol used to preserve five unprocessed freshwater macroinvertebrate bulk samples, in relation to ethanol subsampling day and DNA extraction method

Parametric coefficients	DNA concentration (ng/μl)				DIN value			
	Estimate	*SE*	*t*‐value	*p*	Estimate	*SE*	*t*‐value	*p*
Intersect	3.854	0.617	6.249	4.5 × 10^−09^ ***	4.820	0.446	10.803	<2 × 10^−16^ ***
TISSUE	−1.385	0.814	−1.702	0.091^ns^	−1.136	0.472	−2.404	0.018*
SOIL	−2.230	0.828	−2.695	0.008**	−3.063	0.508	−6.031	1.3 × 10^−08^ ***
**Smooth terms**	***edf***		***F*‐value**	***p***	***edf***		***F*‐value**	***p***
s(day): BEAD	1.695		2.464	0.200^ns^	1.000		1.493	0.224^ns^
s(day): TISSUE	1.000		0.004	0.947^ns^	1.720		14.385	1.6 × 10^−04^ ***
s(day): SOIL	1.000		0.325	0.569^ns^	1.246		0.137	0.829^ns^

Note

Subsampling was conducted at 1, 2, 3, 5, 7 and 14 days after field sampling, and DNA extractions were performed using three methods: BEAD, TISSUE and SOIL as described in Table S1. For each model, we provide the parameter estimates, standard errors (*SE*) and statistical significance of parametric terms, and the effective degrees of freedom (*edf*) and approximate significance of smooth terms. The shape of the smooth terms is provided in Figure 1.

*** *p* < 0.001.

** *p* < 0.01.

* *p* < 0.05.

^ns^ *p *> 0.05.

### 3.2 Sequencing data

**Figure 2:**
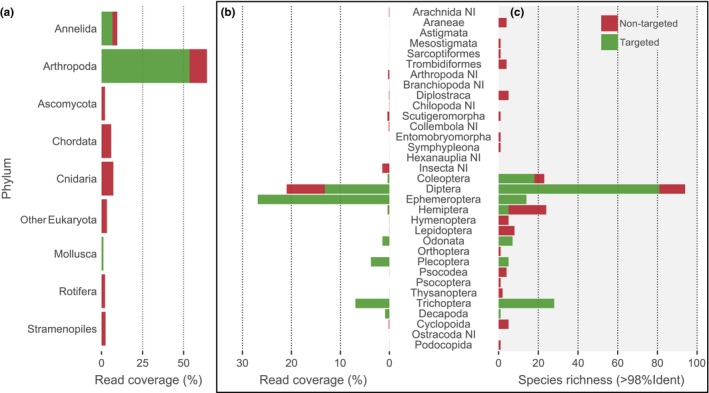
Percentage of read coverage per detected Phyla (a) and Arthropoda Order (b), and taxa richness (≥98% identity) per Arthropoda Order (c), retrieved through metabarcoding of DNA extracted from the 96% ethanol used to preserve five unprocessed samples of freshwater macroinvertebrates. Green bars refer to macroinvertebrate taxa targeted by the Water Framework Directive, and red bars refer to nontarget taxa. NI, not identified [Colour figure can be viewed at http://www.wileyonlinelibrary.com]

Sequencing of all samples generated 12,286,134 reads, with an average read count of 27,303 (±16,501 *SD*) (Table S2). The mean number of reads per sample was similar for the BEAD (27,316), TISSUE (27,844) and SOIL (25,664) extraction methods and varied with subsampling day from a minimum of 25,436 on day 3 to 30,084 on day 7. After sequence curation and cleaning, we obtained 5,357,483 reads (representing 14,997 unique clusters; Table S3), with an average read count of 11,906 (±7,903 *SD*) per sample, of which 91% could be taxonomically assigned at least to Order level and 60% were assigned to species level. Overall, reads were mostly assigned to phylum Arthropoda (64.2%), but there were also other taxa recovered frequently: Annelida (9.6%), Cnidaria (7.2%), Chordata (5.9%), undetermined Eukaryota (3.2%), Stramenopiles (2.4%), Rotifera (2.1%), Ascomycota (2.1%) and Mollusca (1.6%) (Figure 2a, Table S3). Arthropoda orders targeted in WFD were the most represented, namely Ephemeroptera (26.9% reads), Diptera (21.0%), Trichoptera (6.9%) and Plecoptera (3.8%) (Figure 2b). Within Diptera, 7.8% reads could not be assigned to family, and it was uncertain whether they belonged to groups targeted in WFD biomonitoring (Figure 2b). From the 378 taxa assigned to species level, 200 (52.9%) were freshwater macroinvertebrates targeted by the WFD, most of which were Diptera (21.4%), Trichoptera (7.4%) and Coleoptera (4.8%) (Figure 2c).

### 3.3 Taxa richness

**Figure 3:**
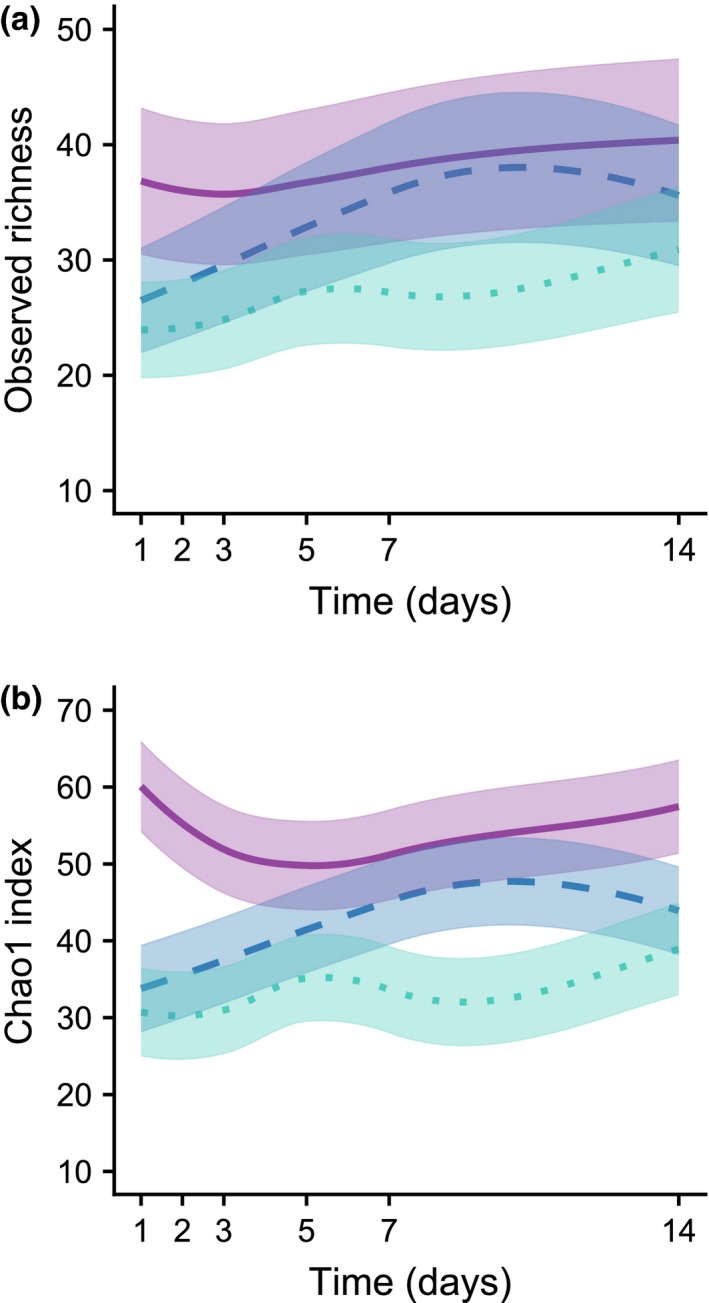
Variation predicted from GAMMs in (a) observed species richness and (b) Chao1 richness estimates assessed through the metabarcoding of DNA extracted from 96% ethanol used to preserve five unprocessed freshwater macroinvertebrate bulk samples, in relation to ethanol subsampling day and DNA extraction method, while controlling for variation in the number of reads across samples. Subsampling was conducted at 1, 2, 3, 5, 7 and 14 days after field sampling, and DNA extractions were performed using three methods: BEAD (purple, solid line), TISSUE (blue, dashed line) and SOIL (green, dotted line). Temporal variation trend lines are provided with the corresponding standard errors. Summary statistics of the GAMMs are provided in Table 2 [Colour figure can be viewed at http://www.wileyonlinelibrary.com]

The mean number of taxa detected per sample was 34 (±13 *SD*) when using the 0.01% threshold for removing rare species, which was smaller than that obtained from the unfiltered matrix (37 ± 14 *SD*) and larger than that using the 0.05% threshold (26 ± 9 *SD*) (Table S2). The mean observed richness increased significantly with the number of reads obtained for the sample, and it was significantly lower in samples extracted using SOIL and TISSUE than using BEAD (Table 2). There were also significant effects of subsampling day on observed richness of samples extracted with the three methods (Table 2). The strongest effect was found for TISSUE, with observed richness increasing markedly up to about 10 days and declining slightly thereafter (Figure 3a), though the later decline may be a model artefact due to lack of data on the interval 7–14 days. For BEAD and SOIL, there was an overall trend for observed richness increasing with subsampling day, though with a small decline in the first three days for BEAD, and small fluctuations over time for SOIL (Figure 3a). It is noteworthy that although BEAD was the method detecting most species in average, the difference in relation to TISSUE was mostly apparent within the first 1–3 days, and largely converged thereafter. Alternative criteria of rare species removal produced broadly similar results, though effects were stronger when using the unfiltered taxa matrix and much weaker when using stricter criteria (Table S4). In particular, when using the 0.03% and 0.05% removal criteria, there were no longer significant differences between BEAD and TISSUE, and no significant effects of subsampling day for BEAD (Table S4). Results using Chao1 were broadly similar, though the decline observed for BEAD in the first few days was stronger for Chao1 than for observed richness estimates (Figure 3b, Table 2).

**Table 2:** Summary statistics of GAMM models relating observed richness and Chao1 richness estimates, to subsampling day and DNA extraction methods

Parametric coefficients	Observed richness				Chao1 richness estimates			
	Estimate	*SE*	*z*‐value	*p*	Estimate	*SE*	*t*‐value	*p*
Intersect	2.602	0.204	12.745	<2 × 10^−16^ ***	−5.934	7.949	−0.747	0.456^ns^
TISSUE	−0.171	0.064	−2.677	0.007**	−14.248	3.784	−3.765	1.9 × 10^−4^ ***
SOIL	−0.327	0.068	−4.785	1.7 × 10^−6^ ***	−19.670	3.898	−5.047	6.8 × 10^−7^ ***
**Smooth terms**	***edf***		***F*‐value**	***p***	***edf***		***F*‐value**	***p***
s(day): BEAD	1		4.252	0.039*	2.335		3.551	0.014*
s(day): TISSUE	2.298		85.497	< 2 × 10^−16^ ***	2.189		16.578	8.1 × 10^−8^ ***
s(day): SOIL	1.297		15.664	9.4 × 10^−4^ ***	1		4.364	0.037*

Note

Extractions were performed from the ethanol used to preserve five unprocessed freshwater macroinvertebrate bulk samples and subsampled on days 1, 2, 3, 5, 7 and 14 after field sampling, using three DNA extraction methods (BEAD, TISSUE and SOIL). For each model, we provide the parameter estimates, standard errors (*SE*) and statistical significance of parametric terms, and the effective degrees of freedom (*edf*) and approximate significance of smooth terms. The shape of the smooth terms is provided in Figure 3. The matrix used to build the models for observed richness excluded rare species with a percentage read count <0.01% from each sample; the unfiltered taxa matrix was used for Chao1 (see Methods); models build using alternative criteria for dealing with observed rare species are given in Table S4.

*** *p* < 0.001.

** *p* < 0.01.

* *p* < 0.05.

^ns^ *p *> 0.05.

### 3.4 Community composition

The PERMANOVA indicated that by far the largest share of variation in the composition of WFD taxa across samples was due to significant differences among sites (64.2%), while differences in coverage among samples had a very small (0.5%), albeit significant contribution to explained variation (Table 3). Although much lower, the subsampling day and the extraction method had significant effects on community variation, though each contributed <2% to explain such variation. In contrast, extraction and PCR replicates did not contribute significantly to explain variation in community composition. The patterns observed were consistent irrespective of the alternative criteria used to deal with rare species (Table S5).

**Table 3:** Summary statistics of nonparametric permutational multivariate analysis of variance (PERMANOVA; 9,999 permutations) for testing the hierarchical contribution of sampling sites (Site), subsampling time (Day), extraction method, extraction replicate and PCR replicate to overall variation in community composition of freshwater macroinvertebrates across sampling units (*n* = 418)

Source of variation	*df*	MSS	*F*	*R* ^2^	*p*
[1] Site	4	20.02	189.39	0.642	0.0001***
[2] Reads	1	0.61	5.793	0.005	0.0001***
[3] Site: Day	5	0.34	3.223	0.014	0.0001***
[4] Site: Day: Extraction method	10	0.23	2.194	0.018	0.0001***
[5] Site: Day: Extraction method: Extraction replicate	10	0.10	0.942	0.008	0.621^ns^
[6] Site: Day: Extraction method: Extraction replicate: PCR replicate	50	0.07	0.644	0.027	1^ns^
Residuals	337	0.11		0.286	
Total	417			1	

Note

The number of reads was also included to control for variation in coverage among samples. For each term, we provide the degrees of freedom (*df*), mean sum of squares (MSS), F model ratio (*F*), r‐squared (*R* ^2^) and *p*‐values. The matrix used to build the model excluded rare species with a percentage read count <0.01% from each sample; models build alternative criteria for dealing with rare species are given in Table S5.

*** *p* < 0.001.

** *p* < 0.01.

* *p* < 0.05.

^ns^ *p *> 0.05.

### 3.5 Differences between metabarcoding and morphology for EPTO

Overall, most EPTO taxa detected morphologically at sampling sites were also detected at the corresponding sites in at least one molecular sampling unit, with similar values for BEAD (78.8% ± 11.0 *SD*; 68.0%–94.1%) and TISSUE (76.4% ± 7.4 *SD*; 70.7%–88.2%), but slightly lower for SOIL (70.4% ± 12.5% *SD*; 54.5%–88.2%) (Table S6). However, about 40%–50% of the EPTO taxa detected through metabarcoding were not detected through morphological identification, either for BEAD (44.6% ± 9.6 *SD*; 34.6%–57.9%), TISSUE (44.5% ± 9.8 *SD*; 32.8%–58.8%) or SOIL (47.9% ± 14.6 *SD*; 28.0%–66.7%) (Table S6).

**Figure 4:**
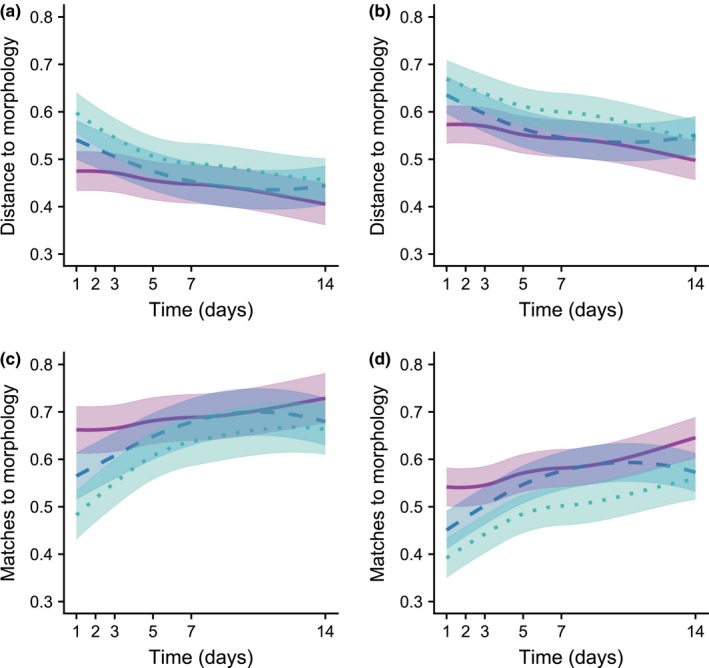
Variation predicted from GAMMs in Jaccard distance (a,b) and percentage matching (c,d) between EPTO community composition estimated from morphological and metabarcoding data, at family (a,c) and species (b,d) levels, in relation to ethanol subsampling day and DNA extraction method, while controlling for variation in the number of reads across samples. Subsampling was conducted at 1, 2, 3, 5, 7 and 14 days after field sampling, and DNA extractions were performed using three methods: BEAD (purple, solid line), TISSUE (blue, dashed line) and SOIL (green, dotted line). Temporal variation trend lines are provided with the corresponding standard errors. Summary statistics of the GAMMs are provided in Table 4 [Colour figure can be viewed at http://www.wileyonlinelibrary.com]

Both at family and species levels, the Jaccard distance index between metabarcoding and morphology of EPTO increased with the read coverage of the samples and it was significantly lower for BEAD than SOIL but not TISSUE (Figure 4a, c; Table 4). Distances declined significantly with subsampling day for all extraction methods. The small increase observed for TISSUE after about the 10th day may be an artefact resulting from the lack of subsampling in days 7 to 14. Results obtained with percentage of matching were qualitatively similar, with SOIL showing a significantly poorer performance than the other two methods, and matching with morphology increasing significantly over time (Figure 4b, d; Table 4). For both the Jaccard distance and percentage matching, the results obtained were similar when using alternative criteria for dealing with rare taxa, though with stronger and more significant results obtained when using unfiltered taxa matrix than when using stricter removal criteria (Tables S7, S8). In particular, significant effects of extraction method disappeared or became very weak when using the 0.03% or 0.05% removal criteria, the same occurring for the temporal trends using BEAD and the 0.05% removal criteria (Tables S7, S8).

**Table 4:** Summary statistics of GAMM models relating Jaccard's distance and percentage matching between the composition of EPTO communities inferred from morphology and metabarcoding, in relation to subsampling day and DNA extraction methods

Jaccard's Distance	Family				Species			
Parametric coefficients	Estimate	*SE*	*z*‐value	*p*	Estimate	*SE*	*t*‐value	*p*
Intersect	0.610	0.058	10.42	<2 × 10^−16^ ***	0.731	0.052	14.123	<2 × 10^−16^ ***
TISSUE	0.034	0.021	1.596	0.111^ns^	0.031	0.019	1.622	0.106^ns^
SOIL	0.067	0.022	2.987	0.003**	0.060	0.020	2.992	0.003**
**Smooth terms**	***edf***		***F*‐value**	***p***	***edf***		***F*‐value**	***p***
s(day): BEAD	1.000		7.411	0.007**	1		13.550	2.6 × 10^−4^ ***
s(day): TISSUE	2.205		21.612	5.5 × 10^−10^ ***	2.455		26.190	3.3 × 10^−12^ ***
s(day): SOIL	2.032		16.302	1.2 × 10^−7^ ***	1.381		31.240	1.5 × 10^−8^ ***

Percentage Matching	Family				Species			
Parametric coefficients	Estimate	*SE*	*z*‐value	*p*	Estimate	*SE*	*t*‐value	*p*
Intersect	0.520	0.068	7.685	1.2 × 10^−13^ ***	0.303	0.057	5.277	2.1 × 10^−7^ ***
TISSUE	−0.052	0.032	−1.634	0.103 ns	−0.048	0.026	−1.839	0.067* ns*
SOIL	−0.098	0.033	−2.997	0.003**	−0.094	0.027	−3.514	4.9 × 10^−4^ ***
**Smooth terms**	***edf***		***F*‐value**	***p***	**edf**		***F*‐value**	***p***
s(day): BEAD	1		5.565	0.019*	1		17.270	3.9 × 10^−5^ ***
s(day): TISSUE	2.315		26.032	2.9 × 10^−12^ ***	2.458		34.130	7.7 × 10^−16^ ***
s(day): SOIL	2.173		23.066	1.2 × 10^−10^ ***	1.822		28.600	4.0 × 10^−9^ ***

Note

Extractions were performed from the ethanol used to preserve five unprocessed freshwater macroinvertebrate bulk samples and subsampled on days 1, 2, 3, 5, 7 and 14 after field sampling, using three DNA extraction methods (BEAD, TISSUE and SOIL). For each model, we provide the parameter estimates, standard errors (*SE*) and statistical significance of parametric terms, and the effective degrees of freedom (*edf*) and approximate significance of smooth terms. The shape of the smooth terms is provided in Figure 4. The matrix used to build the models excluded rare species with a percentage read count <0.01% from each sample; models build using alternative criteria for dealing with rare species are given in Tables S7 and S8.

*** *p* < 0.001.

** *p* < 0.01.

* *p* < 0.05.

^ns^ *p *> 0.05.

## 4 DISCUSSION

Our results confirmed previous studies showing that DNA metabarcoding of 96% ethanol used to preserve freshwater macroinvertebrate bulk samples can provide reliable information on taxa diversity and composition (Hajibabaei et al., 2012; Zizka et al., 2018). We further show that this information can be obtained even from unprocessed bulk samples preserved in the field without sorting, and thus mixed with a wide range of potential contaminants and PCR inhibitors originated from sediments and plant material (Schrader, Schielke, Ellerbroek, & Johne, 2012). Similarly to Zizka et al. (2018), we were able to retrieve relatively high diversity of taxa from a wide range of phyla, across nearly all the samples analysed, with a strong representation of freshwater macroinvertebrate taxa considered in the WFD, the main target of this study. Furthermore, information from metabarcoding clearly detected the ecological signal corresponding to marked variations in the composition of macroinvertebrate communities across sampling sites. However, we also show significant effects of technical variants such as DNA extraction methods and timing of ethanol subsampling in relation to the field bulk collection, which can influence community diversity and composition estimates. Overall, our study suggests that metabarcoding of preservative ethanol from bulk samples may provide a promising tool for cost‐effective biomonitoring programmes of freshwater benthic macroinvertebrates, though care should be taken with the choice and standardization of methods, from extraction to bioinformatic analysis (Leese et al., 2016; Pawlowski et al., 2018; Zizka et al., 2018).

As previously described for other types of samples (Deiner, Walser, Mächler, & Altermatt, 2015; Hermans, Buckley, & Lear, 2018; Majaneva, Diserud, Eagle, Hajibabaei, & Ekrem, 2018), the DNA extraction method was one of the main technical factors affecting metabarcoding community estimates from preservative ethanol. Overall, our results showed that column‐based DNA extraction methods (TISSUE and SOIL) tended to have lower performance compared to the magnetic‐based method (BEAD). In fact, TISSUE and SOIL, particularly the latter, resulted in lower concentrations and integrity of DNA than BEAD, detected less taxa and produced larger dissimilarities in relation to the community composition assessed morphologically. These results were robust to the effect of differences in coverage among samples, which was explicitly controlled statistically by incorporating the number of reads as an offset variable in all models. However, effects were less evident when using stricter criteria for removing rare species with low read counts (i.e. species with a proportion of counts in a sample <0.03% or <0.05%), which suggests that differences between methods were influenced to at least some extent for differential ability to detect rare species. The lower performance of column‐based than magnetic‐based methods was possibly related to the higher probability of DNA extracts to be washed away, while potentially causing higher retention of contaminants (e.g. cell debris, protein, polysaccharides or humic acids) despite the inclusion of an inhibitor removal solution. The performance of SOIL was particularly poor, likely due to the bead‐beating cell lysis step. The majority of DNA in the preservative ethanol might be already in its free form and/or partially degraded, and mechanical lysis can be too damaging for its integrity (Hermans et al., 2018; Leray & Knowlton, 2015), thus affecting the number of taxa recovered. The highest performance of BEAD was probably related with (a) higher affinity of magnetic beads to high‐molecular‐weight genomic DNA, leaving out potential low, degraded DNA present in preservative ethanol; (b) minimize DNA washout; and (c) lower yields of contaminants. Overall, results suggest that variations observed among extraction methods were probably related to differential procedures for cell lysis and DNA capture.

The timing of ethanol subsampling did not have significant effects on DNA concentration, but there was a significant increase in DNA integrity over time when using TISSUE. In contrast, there were marked significant effects of subsampling day on metabarcoding results. Temporal effects were particularly strong for SOIL and TISSUE, with performance increasingly rapidly during the first seven to ten days after field sampling, and levelling‐off or slightly declining thereafter. The later declines, however, could be a model artefact due to the lack of subsampling in days 8 to 13, as well as lack of data beyond day 14. Effects were also marked for SOIL, with a general tendency for increasing performance with subsampling day. Results for BEAD were generally weaker and less consistent than for the other methods, though the highest performance also tended to be obtained for subsampling in the period 7–14 days. These patterns were weaker when using stricter criteria for the removal of taxa with a low proportion of read counts, indicating that they were affected to at least some extent by the ability for detecting more rare species at later subsampling dates. Reasons for these results may derive at least partly from a progressive release of small quantities of DNA from the macroinvertebrates preserved in ethanol, particularly during the first week after field sampling. This release was probably not sufficient to cause appreciable changes in the concentration and integrity of the extracted DNA, but it was likely enough to enhance detection of rarer species and thus increase taxa richness and the similarity between metabarcoding and morphology. These effects were weaker for BEAD probably because it consistently yielded higher DNA concentration and integrity irrespective of subsampling day, and thus likely retrieved DNA from rare species even at low concentrations in the preservative ethanol. In contrast, the two column‐based methods always obtained lower concentrations and integrity of DNA, likely with comparatively higher presence of inhibitors, and thus could only detect rarer species when the concentration of their DNA increased in the preservative ethanol.

Although there were significant effects of extraction method and subsampling day on the estimates of community composition, these effects accounted for about 35 to 45 times less variation than that observed among sampling sites. Furthermore, variation in community composition accounted for by either extraction or PCR replicates was also much smaller compared to variation among sites, and even compared to variation among extraction methods and subsampling day. It should be borne in mind, however, that we selected sites within the same river basin that a priori were expected to have contrasting macroinvertebrate communities due to differences in local habitats, to test the impacts of laboratory procedures in samples reflecting a wide range of ecological conditions. Therefore, the contribution of sampling sites to overall community variation would likely have been lower if we had chosen ecologically more similar sites. Nevertheless, our results suggest that metabarcoding from preservative ethanol can successfully detect at least large variations in community composition among sites, irrespective of the technical alternatives and levels of replication adopted. Finally, it is noteworthy that variation in coverage had a small, albeit significant effect on variation in community composition between samples, which was much smaller than the effect of this variable on species richness estimators.

The percentage of EPTO taxa morphologically identified at sampling sites that were detected through metabarcoding in at least one molecular sample was high, particularly when using BEAD or TISSUE (≈70%–95%). Percentage of matchings, however, were smaller when considering individual subsampling units, particularly at species level, though they were higher in analysis made with BEAD and with samples taken more than seven days after field sampling. The lower matchings in the individual units were probably a consequence of some species having low concentrations of DNA in the ethanol solutions, which were thus difficult to detect systematically due to the relatively small volume of the aliquot used in our study (2 ml). This suggests that larger volumes of ethanol may need to be taken in future studies to detect consistently all the species represented in the bulk (e.g. Zizka et al., 2018). In contrast, Jaccard's distances were relatively high between morphology and metabarcoding, though they also declined when using BEAD and in subsamples taken more than seven days after field sampling. This was because about 40%–50% of taxa detected through metabarcoding were not detected through morphology, irrespective of extraction method. There may be several reasons for metabarcoding missing species detected morphologically, including the lack of a sufficiently extensive DNA barcode reference collection (Elbrecht, Vamos, et al., 2017), which in our case resulted in 40% of reads unassigned to species. Also, primer bias may have caused some taxa to be missed (Elbrecht & Leese, 2017; Elbrecht, Vamos, et al., 2017), while high DNA dilution and degradation in the preservative ethanol may have resulted in the loss of rare taxa during subsampling, extraction or PCR amplification. The latter view is supported by the observation that percentage matching was lower for individual subsamples than for subsamples combined, suggesting that DNA from some taxa was present in some subsamples but not in others. The detection of more species by metabarcoding may also be a consequence of contamination, though care was taken during field and laboratory procedures to avoid it as much as possible. Besides the errors induced by metabarcoding, the patterns obtained can also reflect the limitations and errors of the morphological approach itself, making it difficult to compare our results with those of other studies using mock samples where the mix of species was known without error (Elbrecht & Leese, 2017). In fact, our morphological data, as indeed any comparable data based on field sampling under real conditions, may have missed taxa that were detected with metabarcoding, due for instance to taxa misidentification, the inability to identify some small specimens and larval stages to species or even family levels, or the impossibility to detect eventual taxa represented by specimens that were destroyed or overlooked during the collection, sorting and identification processes (Elbrecht, Vamos, et al., 2017).

Overall, our results provide some guidance on future efforts to develop ecological monitoring programmes for freshwaters based on the metabarcoding of macroinvertebrate bulk samples. First, we confirm that DNA extracted from 96% ethanol used to preserve bulk samples in the field may provide a cost‐effective approach to characterize freshwater macroinvertebrate communities (Zizka et al., 2018), as it avoids the preprocessing steps (e.g. cleaning and sorting) required when undertaking metabarcoding from the bulks themselves (Aylagas et al., 2016; Elbrecht, Peinert, et al., 2017). This should minimize the potential for cross‐contamination, the costs and time required to obtain the data following field sampling, and thereby potentially allowing an increase in the number of sites sampled. Second, we highlight the importance of obtaining comprehensive reference collections of DNA barcodes for target taxa, as this may greatly influence the results (Ekrem, Willassen, & Stur, 2007; Elbrecht, Vamos, et al., 2017). Though this may be particularly important in the case of indicator organisms such as EPTO, less known but highly diverse groups such as Diptera should not be neglected since they yield a large number of unassigned reads thus contributing to uncertainties in the data (Ekrem et al., 2007; Kwong, Srivathsan, & Meier, 2012). Third, we suggest that the DNA extraction method needs to be carefully selected and that magnetic‐based protocols such as BEAD are likely to provide better results than column‐based protocols such as TISSUE and SOIL. This is important because commercial column‐based extraction kits are currently the most commonly used methods in metabarcoding for freshwater bioassessment studies (Andújar et al., 2018; Carew et al., 2013; Deiner et al., 2015; Gibson et al., 2014; Hermans et al., 2018; Linard et al., 2016), thereby requiring further assessment on the potential of magnetic bead technology (e.g. Leontidou et al., 2018; Krehenwinkel et al., 2018), or even other approaches such as the salting‐out protocol (Elbrecht & Steinke, 2019; Elbrecht, Vamos, et al., 2017). Fourth, we suggest that the results of preservative ethanol metabarcoding are significantly improved when subsampling 7–14 days after field collection rather than earlier on, though this is less important when using the more efficient BEAD protocol. Nevertheless, further research is needed on how the timing of subsampling affects metabarcoding results beyond the time frame analysed in our study. Finally, we suggest that when trading‐off biological replication (e.g. the number of sites sampled) and technical replication (e.g. the number of extraction or PCR replicates per site) due for instance to human, logistic and financial limitations, it should be duly considered that the former is often the main source of variation in community composition (Mata et al., 2018; this study), and thus that sampling a large number of sites should be essential to obtain an adequate appreciation of ecological variability in freshwater systems. Future studies should complement our research by further evaluating the impacts of additional methodological procedures, including for instance sample preprocessing (ultrasonic irradiation, shaking, freezing; Zizka et al., 2018) and how it affects subsequent steps in time, ethanol concentrations (Elbrecht, Vamos, et al., 2017; Linard et al., 2016), the ratio of ethanol to bulk volumes and the volume of ethanol analysed. Also, studies are needed on the differential recovery of DNA from different taxa due to variation in body characteristics (e.g. soft vs. hard bodied arthropods), which can affect metabarcoding results (Carew, Coleman, & Hoffmann, 2018; Zizka et al., 2018). These studies should be essential to gain a better understanding of methodological challenges and potential biases throughout the metabarcoding workflow, from the field through the laboratory, to the bioinformatics processing of sequencing data, thereby contributing to standardize protocols to be used in the next‐generation biomonitoring of freshwater ecosystems (Elbrecht, Vamos, et al., 2017; Pawlowski et al., 2018).

## AUTHOR CONTRIBUTIONS

All authors contributed to the research experiment, and reviewed and approved the final manuscript; F.M.S.M., P.B. and A.F.F. designed the research; P.P. conducted the fieldwork; F.M.S.M. conducted the laboratory and bioinformatics analyses with contributions from P.C.A., J.P. and M.G; A.T. conducted the morphological identification; F.M.S.M. and P.B. conducted the data analysis and drafted the manuscript, with contributions from all co‐authors.

## Data Availability

Raw sequencing output is provided as FASTQ files in ENA project Accession no. PRJEB31133, sample Accession nos. ERS3202521‐ERS3203000. All data needed to replicate the analyses reported in this study are provided in the Tables[Link], [Link], [Link], [Link], and are also available at the BioStudies repository (https://www.ebi.ac.uk/biostudies/studies/S-BSST241), Accession no. S‐BSST241.

## References

[men13012-bib-0001] Andújar, C. , Arribas, P. , Gray, C. , Bruce, C. , Woodward, G. , Yu, D. W. , & Vogler, A. P. (2018). Metabarcoding of freshwater invertebrates to detect the effects of a pesticide spill. Molecular Ecology, 27, 146–166. 10.1111/mec.14410 29113023

[men13012-bib-0002] Aylagas, E. , Borja, Á. , Irigoien, X. , & Rodríguez‐Ezpeleta, N. (2016). Benchmarking DNA metabarcoding for biodiversity‐based monitoring and assessment. Frontiers in Marine Science, 3, 96 10.3389/fmars.2016.00096

[men13012-bib-0003] Baird, D. J. , & Hajibabaei, M. (2012). biomonitoring 2.0: A new paradigm in ecosystem assessment made possible by next‐generation DNA sequencing. Molecular Ecology, 21, 2039–2044. 10.1111/j.1365-294X.2012.05519.x 22590728

[men13012-bib-0004] Bálint, M. , Márton, O. , Schatz, M. , Düring, R. A. , & Grossart, H. P. (2018). Proper experimental design requires randomization/balancing of molecular ecology experiments. Ecology and Evolution, 8, 1786–1793. 10.1002/ece3.3687. 29435253 PMC5792580

[men13012-bib-0005] Birk, S. , Bonne, W. , Borja, A. , Brucet, S. , Courrat, A. , Poikane, S. , … Hering, D. (2012). Three hundred ways to assess Europe's surface waters: An almost complete overview of biological methods to implement the Water Framework Directive. Ecological Indicators, 18, 31–41. 10.1016/j.ecolind.2011.10.009

[men13012-bib-0006] Bonada, N. , Prat, N. , Resh, V. H. , & Statzner, B. (2006). Developments in aquatic insect biomonitoring: A comparative analysis of recent approaches. Annual Review of Entomology, 51, 495–523. 10.1146/annurev.ento.51.110104.151124 16332221

[men13012-bib-0007] Boyer, F. , Mercier, C. , Bonin, A. , Le Bras, Y. , Taberlet, P. , & Coissac, E. (2016). obitools: A unix‐inspired software package for DNA metabarcoding. Molecular Ecology Resources, 16, 176–182. 10.1111/1755-0998.12428 25959493

[men13012-bib-0008] Carew, M. E. , Coleman, R. A. , & Hoffmann, A. A. (2018). Can non‐destructive DNA extraction of bulk invertebrate samples be used for metabarcoding? PeerJ, 6, e4980 10.7717/peerj.4980 29915700 PMC6004113

[men13012-bib-0009] Carew, M. E. , Pettigrove, V. J. , Metzeling, L. , & Hoffmann, A. A. (2013). Environmental monitoring using next generation sequencing: Rapid identification of macroinvertebrate bioindicator species. Frontiers in Zoology, 10, 45 10.1186/1742-9994-10-45 23919569 PMC3750358

[men13012-bib-0010] Chao, A. , & Chiu, C. (2016). Nonparametric estimation and comparison of species richness In eLS (pp. 863–11). Chichester: John Wiley & Sons, Ltd.

[men13012-bib-0011] Craig, L. S. , Olden, J. D. , Arthington, A. H. , Entrekin, S. , Hawkins, C. P. , Kelly, J. J. , … Strayer, D. L. (2017). Meeting the challenge of interacting threats in freshwater ecosystems: A call to scientists and managers. Elementa: Science of the Anthropocene, 5, 72 10.1525/elementa.256

[men13012-bib-0012] De Barba, M. , Miquel, C. , Boyer, F. , Mercier, C. , Rioux, D. , Coissac, E. , & Taberlet, P. (2014). DNA metabarcoding multiplexing and validation of data accuracy for diet assessment: application to omnivorous diet. Molecular Ecology Resources, 14, 306–323. 10.1111/1755-0998.12188. 24128180

[men13012-bib-0013] Deiner, K. , Walser, J. C. , Mächler, E. , & Altermatt, F. (2015). Choice of capture and extraction methods affect detection of freshwater biodiversity from environmental DNA. Biological Conservation, 183, 53–63. 10.1016/j.biocon.2014.11.018

[men13012-bib-0014] Ekrem, T. , Willassen, E. , & Stur, E. (2007). A comprehensive DNA sequence library is essential for identification with DNA barcodes. Molecular Phylogenetics and Evolution, 43, 530–542. 10.1016/j.ympev.2006.11.021 17208018

[men13012-bib-0015] Elbrecht, V. , & Leese, F. (2017). Validation and development of COI metabarcoding primers for freshwater macroinvertebrate bioassessment. Frontiers in Environmental Science, 5, 11 10.3389/fenvs.2017.00011

[men13012-bib-0016] Elbrecht, V. , Peinert, B. , & Leese, F. (2017). Sorting things out: Assessing effects of unequal specimen biomass on DNA metabarcoding. Ecology and Evolution, 7, 6918–6926. 10.1002/ece3.3192 28904771 PMC5587478

[men13012-bib-0017] Elbrecht, V. , & Steinke, D. (2019). Scaling up DNA metabarcoding for freshwater macrozoobenthos monitoring. Freshwater Biology, 64, 380–387. 10.1111/fwb.13220

[men13012-bib-0018] Elbrecht, V. , Vamos, E. E. , Meissner, K. , Aroviita, J. , & Leese, F. (2017). Assessing strengths and weaknesses of DNA metabarcoding‐based macroinvertebrate identification for routine stream monitoring. Methods in Ecology and Evolution, 8, 1265–1275. 10.1111/2041-210X.12789

[men13012-bib-0019] Gansauge, M.‐T. , & Meyer, M. (2013). Single‐stranded DNA library preparation for the sequencing of ancient or damaged DNA. Nature Protocols, 8, 737 10.1038/nprot.2013.038 23493070

[men13012-bib-0020] Gibson, J. F. , Shokralla, S. , Curry, C. , Baird, D. J. , Monk, W. A. , King, I. , & Hajibabaei, M. (2015). Large‐scale biomonitoring of remote and threatened ecosystems via high‐throughput sequencing. PLoS One, 10, e0138432 10.1371/journal.pone.0138432 26488407 PMC4619546

[men13012-bib-0021] Gibson, J. , Shokralla, S. , Porter, T. m. , King, I. , van Konynenburg, S. , Janzen, D. H. , … Hajibabaei, M. (2014). Simultaneous assessment of the macrobiome and microbiome in a bulk sample of tropical arthropods through DNA metasystematics. Proceedings of the National Academy of Sciences, 111, 8007–8012. 10.1073/pnas.1406468111 PMC4050544 24808136

[men13012-bib-0022] Hajibabaei, M. , Shokralla, S. , Zhou, X. , Singer, G. A. , & Baird, D. J. (2011). Environmental barcoding: A next‐generation sequencing approach for biomonitoring applications using river benthos. PLoS One, 6, e17497 10.1371/journal.pone.0017497 21533287 PMC3076369

[men13012-bib-0023] Hajibabaei, M. , Spall, J. L. , Shokralla, S. , & van Konynenburg, S. (2012). Assessing biodiversity of a freshwater benthic macroinvertebrate community through non‐destructive environmental barcoding of DNA from preservative ethanol. BMC Ecology, 12, 28 10.1186/1472-6785-12-28 23259585 PMC3542036

[men13012-bib-0024] Hermans, S. M. , Buckley, H. L. , & Lear, G. (2018). Optimal extraction methods for the simultaneous analysis of DNA from diverse organisms and sample types. Molecular Ecology Resources, 18, 557–569. 10.1111/1755-0998.12762 29394525

[men13012-bib-0025] Instituto da Água (INAG) (2008). Manual para a avaliação biológica da qualidade da água em sistemas fluviais segundo a Directiva Quadro da Água: Protocolo de amostragem e análise para os macroinvertebrados bentónicos. Lisboa: Instituto da Água I.P Retrieved from https://www.apambiente.pt/dqa/assets/01-protocolo-de-amostragem-e-an%C3%A1lise-para-os-macroinvertebrados-bent%C3%B3nicos.pdf

[men13012-bib-0026] Kearse, M. , Moir, R. , Wilson, A. , Stones‐Havas, S. , Cheung, M. , Sturrock, S. , … Drummond, A. (2012). Geneious Basic: An integrated and extendable desktop software platform for the organization and analysis of sequence data. Bioinformatics, 28, 1647–1649. 10.1093/bioinformatics/bts199 22543367 PMC3371832

[men13012-bib-0027] Kircher, M. , Sawyer, S. , & Meyer, M. (2011). Double indexing overcomes inaccuracies in multiplex sequencing on the Illumina platform. Nucleic Acids Research, 40, e3 10.1093/nar/gkr771 22021376 PMC3245947

[men13012-bib-0028] Krehenwinkel, H. , Fong, M. , Kennedy, S. , Huang, E. G. , Noriyuki, S. , Cayetano, L. , & Gillespie, R. (2018). The effect of DNA degradation bias in passive sampling devices on metabarcoding studies of arthropod communities and their associated microbiota. PLoS One, 13, e0189188 10.1371/journal.pone.0189188 29304124 PMC5755739

[men13012-bib-0029] Kwong, S. , Srivathsan, A. , & Meier, R. (2012). An update on DNA barcoding: Low species coverage and numerous unidentified sequences. Cladistics, 28, 639–644. 10.1111/j.1096-0031.2012.00408.x 34856738

[men13012-bib-0030] Leese, F. , Altermatt, F. , Bouchez, A. , Ekrem, T. , Hering, D. , Meissner, K. , … Zimmermann, J. (2016). DNAqua‐Net: Developing new genetic tools for bioassessment and monitoring of aquatic ecosystems in Europe. Research Ideas and Outcomes, 2, e11321 10.3897/rio.2.e11321

[men13012-bib-0031] Leontidou, K. , Vernesi, C. , De Groeve, J. , Cristofolini, F. , Vokou, D. , & Cristofori, A. (2018). DNA metabarcoding of airborne pollen: New protocols for improved taxonomic identification of environmental samples. Aerobiologia, 34, 63–74. 10.1007/s10453-017-9497-z

[men13012-bib-0032] Leray, M. , & Knowlton, N. (2015). DNA barcoding and metabarcoding of standardized samples reveal patterns of marine benthic diversity. Proceedings of the National Academy of Sciences, 112, 2076–2081. 10.1073/pnas.1424997112 PMC4343139 25646458

[men13012-bib-0033] Linard, B. , Arribas, P. , Andújar, C. , Crampton‐Platt, A. , & Vogler, A. P. (2016). Lessons from genome skimming of arthropod‐preserving ethanol. Molecular Ecology Resources, 16, 1365–1377. 10.1111/1755-0998.12539 27235167

[men13012-bib-0034] Majaneva, M. , Diserud, O. H. , Eagle, S. H. , Hajibabaei, M. , & Ekrem, T. (2018). Choice of DNA extraction method affects DNA metabarcoding of unsorted invertebrate bulk samples. Metabarcoding and Metagenomics, 2, e26664 10.3897/mbmg.2.26664

[men13012-bib-0035] Mata, V. A. , Rebelo, H. , Amorim, F. , McCracken, G. F. , Jarman, S. , & Beja, P. (2018). How much is enough? Effects of technical and biological replication on metabarcoding dietary analysis. Molecular Ecology, 28, 165–175. 10.1111/mec.14779 29940083 PMC7379978

[men13012-bib-0036] McMurdie, P. J. , & Holmes, S. (2013). phyloseq: An R package for reproducible interactive analysis and graphics of microbiome census data. PLoS One, 8, e61217 10.1371/journal.pone.0061217 23630581 PMC3632530

[men13012-bib-0037] McMurdie, P. J. , & Holmes, S. (2014). Waste not, want not: Why rarefying microbiome data is inadmissible. PLoS Computational Biology, 10, e1003531 10.1371/journal.pcbi.1003531 24699258 PMC3974642

[men13012-bib-0038] Nichols, R. V. , Vollmers, C. , Newsom, L. A. , Wang, Y. , Heintzman, P. D. , Leighton, M. K. , … Shapiro, B. (2018). Minimizing polymerase biases in metabarcoding. Molecular Ecology Resources, 18, 927–939. 10.1111/1755-0998.12895 29797549

[men13012-bib-0039] Oksanen, J. , Blanchet, G. , Friendly, M. , Kindt, R. , Legendre, P. , McGlinn, D. , Wagner, H. (2018). vegan: Community ecology package. R package version 2.4‐6. https://CRAN.R-project.org/package=vegan

[men13012-bib-0040] Pawlowski, J. , Kelly‐Quinn, M. , Altermatt, F. , Apothéloz‐Perret‐Gentil, L. , Beja, P. , Boggero, A. , … Kahlert, M. (2018). The future of biotic indices in the ecogenomic era: Integrating DNA metabarcoding in biological assessment of aquatic ecosystems. Science of the Total Environment, 637, 1295–1310. 10.1016/j.scitotenv.2018.05.002 29801222

[men13012-bib-0041] Schielzeth, H. , & Nakagawa, S. (2013). Nested by design: Model fitting and interpretation in a mixed model era. Methods in Ecology and Evolution, 4, 14–24. 10.1111/j.2041-210x.2012.00251.x

[men13012-bib-0042] Schrader, C. , Schielke, A. , Ellerbroek, L. , & Johne, R. (2012). PCR inhibitors–occurrence, properties and removal. Journal of Applied Microbiology, 113, 1014–1026. 10.1111/j.1365-2672.2012.05384.x 22747964

[men13012-bib-0043] Sharpton, T. J. , Riesenfeld, S. J. , Kembel, S. W. , Ladau, J. , O'Dwyer, J. P. , Green, J. L. , … Pollard, K. S. (2011). PhylOTU: A high‐throughput procedure quantifies microbial community diversity and resolves novel taxa from metagenomic data. PLoS Computational Biology, 7, e1001061 10.1371/journal.pcbi.1001061 21283775 PMC3024254

[men13012-bib-0044] Shokralla, S. , Porter, T. M. , Gibson, J. F. , Dobosz, R. , Janzen, D. H. , Hallwachs, W. , … Hajibabaei, M. (2015). Massively parallel multiplex DNA sequencing for specimen identification using an Illumina MiSeq platform. Scientific Reports, 5, 9687 10.1038/srep09687 25884109 PMC4401116

[men13012-bib-0045] Shokralla, S. , Singer, G. A. , & Hajibabaei, M. (2010). Direct PCR amplification and sequencing of specimens' DNA from preservative ethanol. BioTechniques, 48, 233 10.2144/000113362 20359306

[men13012-bib-0046] Stein, E. D. , White, B. P. , Mazor, R. D. , Miller, P. E. , & Pilgrim, E. M. (2013). Evaluating ethanol‐based sample preservation to facilitate use of DNA barcoding in routine freshwater biomonitoring programs using benthic macroinvertebrates. PLoS One, 8, e51273 10.1371/journal.pone.0051273 23308097 PMC3537618

[men13012-bib-0047] Sweeney, B. W. , Battle, J. M. , Jackson, J. K. , & Dapkey, T. (2011). Can DNA barcodes of stream macroinvertebrates improve descriptions of community structure and water quality? Journal of the North American Benthological Society, 30, 195–216. 10.1899/10-016.1

[men13012-bib-0048] Taberlet, P. , Bonin, A. , Zinger, L. , & Coissac, E. (2018). Environmental DNA for biodiversity research and monitoring (p. 253). Oxford: Oxford University Press.

[men13012-bib-0049] Taberlet, P. , Coissac, E. , Pompanon, F. , Brochmann, C. , & Willerslev, E. (2012). Towards next‐generation biodiversity assessment using DNA metabarcoding. Molecular Ecology, 21, 2045–2050. 10.1111/j.1365-294X.2012.05470.x 22486824

[men13012-bib-0050] Vörösmarty, C. J. , McIntyre, P. B. , Gessner, M. O. , Dudgeon, D. , Prusevich, A. , Green, P. , … Davies, P. M. (2010). Global threats to human water security and river biodiversity. Nature, 467, 555 10.1038/nature09440 20882010

[men13012-bib-0051] Wickham, H. (2016). ggplot2: Elegant graphics for data analysis (2nd ed.). Cham: Springer 10.1007/978-3-319-24277-4

[men13012-bib-0052] Wood, S. N. (2006). Generalized additive models: An introduction with R (2nd ed.). Boca Raton, MA: CRC Press.

[men13012-bib-0053] Wood, S. , & Scheipl, F. (2017). gamm4: generalized additive mixed models using 'mgcv' and 'lme4'. R package version 0.2‐5. https://CRAN.R-project.org/package=gamm4

[men13012-bib-0054] Zizka, V. M. , Leese, F. , Peinert, B. , & Geiger, M. F. (2018). DNA metabarcoding from sample fixative as a quick and voucher preserving biodiversity assessment method. Genome, 10.1139/gen-2018-0048 30457888

