# Supplementary material for: Have the cake and eat it: Optimizing nondestructive DNA metabarcoding of macroinvertebrate samples for freshwater biomonitoring
Source: Mol Ecol Resour. 2019 Apr 29;19(4):863–76. doi: 10.1111/1755-0998.13012 (PMC6850371; doi:10.1111/1755-0998.13012)
Supplement: Supplementary file 1 [file MEN-19-863-s001.pdf]

MOLECULAR ECOLOGY RESOURCES

Supplemental Information for:

Have the cake and eat it: Optimising non-destructive DNA metabarcoding of macroinvertebrate samples for freshwater bioassessments

Filipa M.S. Martins, Mafalda Galhardo, Ana F. Filipe, Amílcar Teixeira, Paulo Pinheiro, Joana Paupério, Paulo C. Alves, Pedro Beja

Table of Contents:

**Supplementary Methods**	Page 2
**Supplementary Tables**	Page 8
**Supplementary Figures**	Page 21

MOLECULAR ECOLOGY RESOURCES

Supplementary Methods

LABORATORY PROCEDURES

Initial sample preparation

Bulk samples were preserved in 96% ethanol, with an approximate ethanol:bulk ratio of 3:1, and kept at room temperature until ethanol subsampling. Prior to ethanol subsampling, bulk samples were first shaken manually around 15 times. Then 2 mL of ethanol were transferred to five cryotubes of 2 mL (Sarstedt) previously prepared and labelled, and kept at -20ºC until DNA extraction. Ethanol subsampling was performed within a fume hood and precautions were taken to reduce (cross-) contamination risk (UV bench sterilization, mask and cap usage, glove changing between samples, disposable lab coats, filtered tips). Before DNA extraction, cryotubes were opened and placed in an Eppendorf vacuum concentrator at 30ºC overnight using the V-AL option, to assure the evaporation of ethanol.

DNA Extraction Procedures

DNA extraction was carried out from each ethanol sample using the three methods described below and summarised in Table S1.

Column-based mechanical protocol (SOIL)

Samples were prepared using the SL1 Buffer without Enhancer SX and following the NucleoSpin® Soil manufacturer's protocol (MACHEREY-NAGEL GmbH & Co, Düren, Germany). This protocol uses ceramic beads to break up the cells (mechanic lysis) and Inhibitor Removal Columns to eliminate humic substances (including humic acids). At the elution step, the spin columns were placed in Eppendorf Low-Bind tubes, to minimise DNA losses, and incubated with 70 µL of elution buffer for 5 min at room temperature before the final centrifuge. Extractions using NucleoSpin® Soil kit were performed in sets of 24 samples (including one extraction negative) using a vacuum manifold (Qiagen® QIAvac 24 Plus) to reduce the risk of contamination across samples.

Column-based enzymatic protocol (TISSUE)

Samples were prepared following the E.Z.N.A.® Tissue DNA Kit manufacturer's protocol (Omega Bio-tek, Inc., Georgia, United States), but using 300 µL Qiagen® Inhibitex Buffer instead of the manufacturer's lysis buffer (TL Buffer), in order to remove inhibitory compounds. This protocol uses proteinase K to break up the cells (enzymatic lysis). At the elution step, the spin columns were placed in Eppendorf Low-Bind tubes and incubated with 70 µL of elution buffer for 5 min at room temperature before the final centrifuge. Extractions using E.Z.N.A kit were also performed in sets of 24 samples (including one extraction negative) using a vacuum manifold (Qiagen® QIAvac 24 Plus) to reduce the risk of contamination across samples.

Magnetic-based enzymatic protocol (BEAD)

We optimised a new protocol based on magnetic bead technology, enzymatic lysis and Qiagen® Buffer solutions. Samples were digested using 300 µL ATL Buffer and 20 µL Proteinase K, incubating for 3h at 56ºC in a thermoblock. Afterwards, 300 µL AL Solution was added to each tube, manually mixed by inverting and kept for 2min at room temperature. Lysate was transferred to a new 2-ml tube prefilled with 300 µL Isopropanol and 25 µL Agencourt®AMPure®XP beads suspension (A Beckman Coulter Company, Massachusetts, United States), and well resuspended by vortexing. Tubes were placed on a magnetic rack for 5min and the supernatant was removed using a pipette, leaving beads with DNA on the bottom of the tube. Then, 500 µL AW1 Buffer was added and the magnetic beads were resuspended by vortexing. Tubes were placed on the magnetic rack for 5min for the magnet to collect the beads, and then supernatant was completely discarded using a pipette. Tubes were removed from the rack and 500 µL AW2 Buffer was added. Beads were again resuspended by vortexing and the tubes were placed on the magnet for 5min. The supernatant was completely removed using a pipette and, immediately after, 200 µL Ethanol 70% was added to the tubes, still placed in the rack, and kept for 30s. The supernatant was removed with a pipette and the sample air-dried for 5min, making sure that all the ethanol was completely evaporated. Tubes were removed from the magnetic rack and 70 µL Elution Buffer was added. Beads were resuspended by vortexing and incubated at 55°C for 5min, mixing occasionally. Finally, after 5min in the magnetic rack, all supernatant was transferred from tubes to Eppendorf Low-Bind tubes and stored at -20ºC until PCR amplification. Extractions using beads were performed in sets of 16 (including one extraction negative), using 8-tube magnetic racks.

PCR amplification

PCR amplifications were performed using the reverse primer BR2 (Elbrecht & Leese, 2017) and a newly designed forward primer (Martins-2018-COI_Fw, 5´-GGNTGAACHGTHTAYCCHCC-3´), located at the same position as the reverse primer Ill_C_R primer (Shokralla et al., 2015), but less degenerated and reversed complemented. It was after an *in silico* inspection of the available primer sets (Elbrecht & Leese, 2017) against a private reference database (IBI, see below) that we found the need to use a primer enhancing PCR amplification for the EPTO group (Ephemeroptera, Plecoptera, Trichoptera and Odonata), with a special focus on the highly diverse order of Trichoptera. DNA extractions, PCR amplifications and library preparation were performed in physically distinct rooms to avoid contaminations.

BIOINFORMATIC ANALYSIS

DNA sequence handling, curation and cleaning

DNA sequence data were processed using the OBITools program suite (Boyer et al., 2016). The R1/R2 fastq files were retrieved for each sample from Illumina BaseSpace and merged using 'illuminapairedend'. The distribution of alignment scores was assessed using the 'obistat' command, and any consensus sequence with a quality score < 50 was filtered out. Once the full amplicon sequences were built from forward and reverse paired-end reads, each sequence was assigned to the respective sample according to the primer set used to amplify the fragment using 'ngsfilter'. Then, sequences from all samples were concatenated and dereplicated using 'obiuniq -m', annotating sequence headers with the number of reads of each sequence in each sample. Sequences were then curated using 'obigrep' to remove obvious artifacts (Taberlet et al., 2018), discarding sequences with a length outside the expected metabarcode size (310-316), and eliminating sequences occurring just once across the dataset (i.e., singleton reads).

To further filter out noisy sequences and to minimise artefactual inflation of biodiversity estimates, we used 'obiclean' to eliminate those erroneous sequences compatible with indel or substitution errors, based on their lower frequency of occurrence and similarity to most common sequences (de Barba et al., 2014, Taberlet et al., 2018). 'obiclean' builds a directed acyclic graph (DAG), where nodes are sequences and sequences differing by a prespecified number of positions are linked by an arrow, which is directed from the most abundant variant to the rarest one (de Barba et al., 2014). Summits of the DAG are labelled 'head', corresponding to true sequences/abundant PCR products. Rare sequences linked to the 'head' and with a prespecified threshold ratio between counts (rare/abundant counts) are labelled 'internal', corresponding to PCR substitution/indel errors, and sequences not related in the graph to any other are labelled 'singleton', corresponding to rare true sequences or chimeras (de Barba et al., 2014). In our application (obiclean -H -r 0.05), we used the default of one base difference to link sequences and defined a 5% threshold for the abundance ratio to label a sequence as 'internal' of a 'head' variant.

A table with the counts per sample of each sequence retained after 'obiclean' processing was produced using the 'obitab' command, including a total of 473,571 unique cluster sequences. Then, read counts in each sample were normalized to their total sum, and sequences with ≤0.03% of read coverage in at least one sample and ≤5 reads were discarded. Extraction and PCR negative controls were used to filter out potential contaminants, by subtracting the maximum count of a sequence found in a negative control from its count in every sample, and setting its value to zero

if the subtraction led to a negative number. Cluster sequences left with no reads across all samples were discarded. Also, we only retained cluster sequences with 'head' status in at least one sample, and sequences for which their frequency of occurrence in 'singleton' status was higher than in 'internal' status across samples (Bálint et al. 2018). After this final cleaning step, a total of 14,997 unique cluster sequences were kept for subsequent analysis.

Taxonomic assignment per reference database

Each cluster sequence retained after the curation and cleaning processes was assigned to the lowest possible taxon against three reference databases:

- NCBI. The reference nucleotide database was downloaded from NCBI in September 2017 using the BLAST+ command line utilities (Camacho, 2009), version 2.2.31. Sequences were searched against the reference Nucleotide database, using the 'blastn' tool with the 'megablast' option to optimize for highly identical sequences and reporting a maximum of 100 hits per query sequence. The tabular output (6) was selected with additional fields: query length, subject length, subject all titles, subject tax ids, subject scientific names, subject BLAST names, subject common names, subject super kingdoms, query coverage per subject (for all HSPs), query coverage per HSP (alignments).
- BOLD. In order to get access to the private sequences, manual queries were done on the BOLD website (September 2017) using a maximum of 500 query sequences at a time. Result links were parsed using Bold Retriever (bold-retriever.readthedocs.io) (Vesterinen et al., 2016).
- IBI (CIBIO-InBIO Barcoding Initiative). The private invertebrate reference collection IBI was used for performing local searches using the BLAST+ command line utilities (Camacho, 2009), version 2.2.31. First, IBI was converted into a BLAST database using the 'makeblastdb' utility (version 2.6.0+) with default settings. The 'blastn' utility was then used to search against the database with similar settings as described above for the reference Nucleotide database.

Taxonomic assignments were done in separate for each database. For each cluster sequence, we retained assignments with percentage of identity ≥85%, alignment length ≥280bp (90% of expected length), and records 2% below the top hit. Then, sequences were assigned to the species level if there was a top hit with a given species represented in the database with a percentage of identity ≥98%. If there were more than one species with similar percentage identities, the sequence was assigned to a species pair or to a higher taxonomic category shared by the top hit species (e.g., genus or family). Likewise, when percentage identities were <98%, the sequence was assigned to the taxonomic level that was shared by the top hitting taxa,

considering the following identity thresholds: ≥95% for Genus; ≥92% for Family; and ≥90% for Order.

Assignments produced by the three databases were cross-checked, and the best taxonomic assignment was selected for each sequence. Specifically, we retained the assignment with the highest percentage identity at the lowest possible taxonomic level. Frequently, results obtained from IBI were retained, because the database included a large number of taxa collected close to the study area or in other regions of Portugal. However, species-level identifications were often impossible even using the IBI database due to gaps in coverage for many groups, and so identifications at higher taxonomic groups were retained. All taxonomic assignments were manually checked and validated based on the closest hits, and considering also ancillary information such as species geographic ranges and occurrence in the country.

In the case of macroinvertebrate taxa targeted by the Water Framework Directive in Portugal (i.e., species with aquatic life stages of the Orders Turbellaria, Gastropoda, Bivalvia, Oligochaeta, Hirudinea, Crustacea, and Insecta; INAG, 2008), a tree-based approach was used to classify unassigned sequences to species level into phylogenetic distinct taxonomic units (phylOTU; Sharpton et al., 2011). To do this, sequences assigned to the same Order were aligned and clustered hierarchically using Unweighted Pair Group Method with Arithmetic Mean (UPGMA) trees based on HKY distance model (1,000 bootstrap replicates), in Geneious v10 (https://www.geneious.com, Kearse et al., 2012). We then considered a distinct phylOTU each cluster of sequences that was separated from all other clusters by a distance ≥5%, except in the case of the Trichoptera and Hemiptera for which we selected a threshold of 3%. Distance thresholds were estimated from UPGMA trees using both read data and reference data of target species (IBI and public databases). Each phylOTU was then designated by joining the Order, Family or Genus name to which it had been assigned, with the particle 'sp. #' (e.g., sp.1, sp2, etc.) that could note in principle a unique, unknown species. In subsequent analysis estimating the diversity and community composition of WFD taxa we combined species and phylOTU data (≥98% matching). The WFD species matrix was created by merging the read counts of clusters assigned into the same species/phylOTUs using the R phyloseq package (McMurdie & Holmes, 2013).

Since rare taxa can result for instance from cross-contamination or tag jumps during the process (Taberlet et al. 2018), species/phylOTUs with a read coverage <0.01% in each sample were removed. Because the criteria and thresholds to remove rare taxa can influence results, analysis were repeated with the unfiltered taxa matrix, excluding 'singleton' taxa from the matrix (i.e.,

taxa with only one read), and with the matrix trimmed at 0.03% and 0.05% thresholds. Results are presented considering the 0.01% threshold except where indicated otherwise.

REFERENCES

Bálint, M., Márton, O., Schatz, M., Düring, R. A., & Grossart, H. P. (2018). Proper experimental design requires randomization/balancing of molecular ecology experiments. *Ecology and Evolution*, *8*, 1786-1793.

Boyer, F., Mercier, C., Bonin, A., Le Bras, Y., Taberlet, P., & Coissac, E. (2015). obitools: A unix-inspired software package for DNA metabarcoding. *Molecular Ecology Resources*, *16*, 176–182.

Camacho, C., Coulouris, G., Avagyan, V., Ma, N., Papadopoulos, J., Bealer, K., & Madden, T. L. (2009). BLAST+: architecture and applications. *BMC Bioinformatics*, *10*, 421.

Elbrecht, V., & Leese, F. (2017). Validation and development of COI metabarcoding primers for freshwater macroinvertebrate bioassessment. *Frontiers in Environmental Science*, *5*, 11.

Kearse, M., Moir, R., Wilson, A., Stones-Havas, S., Cheung, M., Sturrock, S., … Drummond, A. (2012). Geneious Basic: an integrated and extendable desktop software platform for the organization and analysis of sequence data. *Bioinformatics*, *28*, 1647–1649.

McMurdie, P. J., & Holmes, S. (2013). phyloseq: an R package for reproducible interactive analysis and graphics of microbiome census data. *PLoS ONE*, *8*, e61217.

Oksanen J., Blanchet F. G., Friendly M., Kindt R., Legendre P., McGlinn D., ... Wagner H. (2018). vegan: community ecology package. R package version 2.5-2. http://CRAN.R-project.org/package=vegan

Sharpton, T. J., Riesenfeld, S. J., Kembel, S. W., Ladau, J., O'Dwyer, J. P., Green, J. L., ... Pollard, K. S. (2011). PhylOTU: a high-throughput procedure quantifies microbial community diversity and resolves novel taxa from metagenomic data. *PLoS Computational Biology*, *7*, e1001061.

Vesterinen, E. J., Ruokolainen, L., Wahlberg, N., Peña, C., Roslin, T., Laine, V. N., ... Lilley, T. M. (2016). What you need is what you eat? Prey selection by the bat *Myotis daubentonii*. *Molecular Ecology*, *25*, 1581-1594.

Supplementary Tables

TABLE S1 Summary description of the three different DNA extraction methods used in this study.

	BEAD	TISSUE	SOIL
Cell lysis	enzymatic (pK)	enzymatic (pK)	mechanical
DNA precipitation	ligation solution (Qiagen®BL)	ligation solution (manufac.)	ligation solution (manufac.)
DNA capture	magnetic bead	column	column
Contaminant removal	no	yes (Qiagen®Inhibitex)	yes (manufacturer)
DNA Yield (approx.)	>3 µg	3-25 µg	2-10 µg
Elution volume	70 µL	70 µL	70 µL

MOLECULAR ECOLOGY RESOURCES

TABLE S2 (EXCEL File) Data for each sampling site, including sample ID, sampling site, ethanol subsampling day, extraction method, extraction and PCR replicate, DNA concentration and integrity, sequencing depth and read coverage for different groups of taxa, richness estimates, and parameters describing differences between metabarcoding and morphology for EPTO taxa. Community parameters were estimated considering five alternative criteria to deal with rare taxa in each sample: raw data (no taxa excluded); removal of singletons; and removal of taxa with percentages of read counts <0.01%, <0.03% or <0.05%. NA = Data not available. These data can be used directly to replicate analysis provided in Tables 1, 2 and 4, and in Figures 1, 3 and 4.

TABLE S3 (EXCEL File) Data on taxonomic composition retrieved through metabarcoding for each sample, including the raw number of reads obtained per taxa. These data can be used to replicate all analysis reported in this paper after adequate pre-processing (e.g., trimming of data according to alternative criteria to deal with rare taxa in each sample).

MOLECULAR ECOLOGY RESOURCES

Table S4. Summary statistics of GAMM models relating observed richness estimates to subsampling day and DNA extraction methods. Extractions were performed from the ethanol used to preserve five unprocessed freshwater macroinvertebrate bulk samples and subsampled on days 1, 2, 3, 5, 7 and 14 after field sampling, using three DNA extraction methods (BEAD, TISSUE and SOIL). For each model, we provide the parameter estimates, standard errors (SE) and statistical significance of parametric terms, and the effective degrees of freedom (edf) and approximate significance of smooth terms. Different models were built considering alternative criteria for dealing with rare species (see the main text for details).

**Dataset using the raw data**				
Parametric coefficients	*Estimate*	*SE*	*t-value*	*P*
Intersect	2.203	0.197	11.160	$<2.0\times10^{-16}$***
TISSUE	-0.233	0.080	-2.907	0.004 **
SOIL	-0.395	0.084	-4.725	2.3×10^{-06} ***
log(Reads)	0.181	0.013	14.225	$<2.0\times10^{-16}$***
Smooth terms	*edf*		*F-value*	*P*
s(day):BEAD	1		2.481	0.115 *ns*
s(day):TISSUE	2.282		81.729	$<2.0\times10^{-16}$***
s(day):SOIL	1		12.049	5.2×10^{-04} ***
**Dataset excluding singletons from each sample**				
Parametric coefficients	*Estimate*	*SE*	*t-value*	*P*
Intersect	1.744	0.212	8.218	$<2.0\times10^{-16}$***
TISSUE	-0.139	0.077	-1.818	0.069 *ns*
SOIL	-0.311	0.081	-3.854	1.2×10^{-04} ***
log(Reads)	0.209	0.014	15.227	$<2.0\times10^{-16}$***
Smooth terms	*edf*		*F-value*	*P*
s(day):BEAD	1		2.355	0.125 *ns*
s(day):TISSUE	2.196		73.967	7.8×10^{-16} ***
s(day):SOIL	1.72		20.834	0.001 **
**Dataset excluding from each sample the taxa with a percentage of read counts <0.01%**				
Parametric coefficients	*Estimate*	*SE*	*t-value*	*P*
Intersect	2.602	0.204	12.745	$<2.0\times10^{-16}$***
TISSUE	-0.171	0.064	-2.677	0.007 **
SOIL	-0.327	0.068	-4.785	1.7×10^{-06} ***
log(Reads)	0.117	0.013	9.065	$<2.0\times10^{-16}$***
Smooth terms	*edf*		*F-value*	*P*
s(day):BEAD	1		4.252	0.039*
s(day):TISSUE	2.298		85.497	$<2.0\times10^{-16}$***
s(day):SOIL	1.297		15.664	9.4×10^{-04} ***

****P*<0.001, ***P*<0.01, **P*<0.05, *ns P*>0.05.

TABLE S4 (cont.)

**Dataset excluding from each sample the taxa with a percentage of read counts <0.03%**				
Parametric coefficients	*Estimate*	*SE*	*t-value*	*P*
Intersect	2.592	0.202	12.835	$<2.0\times10^{-16}$***
TISSUE	-0.007	0.060	-0.116	0.908 *ns*
SOIL	-0.199	0.065	-3.035	0.002 **
log(Reads)	0.087	0.014	6.370	1.9×10^{-10} ***
Smooth terms	*edf*		*F-value*	*P*
s(day):BEAD	1		2.280	0.131 *ns*
s(day):TISSUE	2.154		63.200	8.8×10^{-14} ***
s(day):SOIL	1.726		21.700	8.0×10^{-04} ***

**Dataset excluding from each sample the taxa with a percentage of read counts <0.05%**				
Parametric coefficients	*Estimate*	*SE*	*t-value*	*P*
Intersect	2.629	0.196	13.424	$<2.0\times10^{-16}$***
TISSUE	0.076	0.051	1.495	0.135 *ns*
SOIL	-0.128	0.058	-2.204	0.028 *
log(Reads)	0.064	0.014	4.487	7.2×10^{-06} ***
Smooth terms	*edf*		*F-value*	*P*
s(day):BEAD	1		0.488	0.485 *ns*
s(day):TISSUE	2.181		48.755	1.1×10^{-10} ***
s(day):SOIL	1.490		18.326	0.001**

***P<0.001, **P<0.01, *P<0.05, *ns* P>0.05.

MOLECULAR ECOLOGY
RESOURCES

TABLE S5 Summary statistics of non-parametric permutational multivariate analysis of variance (PERMANOVA; 9,999 permutations) for testing the nested contribution of sampling sites (Site), subsampling time (Day), extraction method, extraction replicate, and PCR replicate to overall variation in community composition of freshwater macroinvertebrates across sampling units (n=418). The number of reads was also included to control for variation in coverage among samples. For each term we provide the degrees of freedom (df), mean sum of squares (MSS), F model ratio (F), r-squared (R^2) and *P*-values. Different models were built considering alternative criteria for dealing with rare species (see the main text for details).

**Dataset using the raw data**					
Source of variation	*df*	*MSS*	*F*	R^2	*P*
[1] Site	4	17.54	149.863	0.584	0.0001***
[2] Reads	1	1.02	8.701	0.008	0.0001***
[3] Site:Day	5	0.34	2.937	0.014	0.0001***
[4] Site:Day:Extraction method	10	0.28	2.426	0.024	0.0001***
[5] Site:Day:Extraction method:Extraction replicate	10	0.11	0.907	0.009	0.732 *ns*
[6] Site:Day:Extraction method:Extraction replicate:PCR replicate	50	0.08	0.675	0.033	1 *ns*
Residuals	337	0.12		0.328	
Total	417			1	
**Dataset excluding singletons from each sample**					
Source of variation	*df*	*MSS*	*F*	R^2	*P*
[1] Site	4	20.48	193.863	0.644	0.0001***
[2] Reads	1	1.28	12.070	0.010	0.0001***
[3] Site:Day	5	0.34	3.217	0.013	0.0001***
[4] Site:Day:Extraction method	10	0.23	2.193	0.018	0.0001***
[5] Site:Day:Extraction method:Extraction replicate	10	0.10	0.956	0.008	0.583 *ns*
[6] Site:Day:Extraction method:Extraction replicate:PCR replicate	50	0.07	0.643	0.027	1 *ns*
Residuals	337	0.11		0.280	
Total	417			1	
**Dataset excluding from each sample the taxa with a percentage of read counts <0.01%**					
Source of variation	*df*	*MSS*	*F*	R^2	*P*
[1] Site	4	20.02	189.389	0.642	0.0001***
[2] Reads	1	0.61	5.793	0.005	0.0001***
[3] Site:Day	5	0.34	3.223	0.014	0.0001***
[4] Site:Day:Extraction method	10	0.23	2.194	0.019	0.0001***
[5] Site:Day:Extraction method:Extraction replicate	10	0.10	0.942	0.008	0.621 *ns*
[6] Site:Day:Extraction method:Extraction replicate:PCR replicate	50	0.07	0.644	0.027	1 *ns*
Residuals	337	0.11		0.286	
Total	417			1	

***P<0.001, **P<0.01, *P<0.05, *ns* P>0.05.

MOLECULAR ECOLOGY RESOURCES

TABLE S5 (cont.)

**Dataset excluding from each sample the taxa with a percentage of read counts <0.03%**					
Source of variation	*df*	*MSS*	*F*	R^2	*P*
[1] Site	4	21.54	213.853	0.671	0.0001***
[2] Reads	1	0.40	3.959	0.003	0.0018**
[3] Site:Day	5	0.33	3.260	0.013	0.0001***
[4] Site:Day:Extraction method	10	0.21	2.102	0.016	0.0001***
[5] Site:Day:Extraction method:Extraction replicate	10	0.09	0.929	0.007	0.6452 *ns*
[6] Site:Day:Extraction method:Extraction replicate:PCR replicate	50	0.06	0.638	0.025	1 *ns*
Residuals	337	0.10		0.264	
Total	417			1	
**Dataset excluding from each sample the taxa with a percentage of read counts <0.05%**					
Source of variation	*df*	*MSS*	*F*	R^2	*P*
[1] Site	4	22.16	222.897	0.680	0.0001***
[2] Reads	1	0.30	3.052	0.002	0.0066**
[3] Site:Day	5	0.30	3.006	0.011	0.0001***
[4] Site:Day:Extraction method	10	0.25	2.469	0.019	0.0001***
[5] Site:Day:Extraction method:Extraction replicate	10	0.09	0.940	0.007	0.6181 *ns*
[6] Site:Day:Extraction method:Extraction replicate:PCR replicate	50	0.06	0.595	0.023	1 *ns*
Residuals	337	0.10		0.257	
Total	417			1	

***P<0.001, **P<0.01, *P<0.05, *ns* P>0.05.

MOLECULAR ECOLOGY
RESOURCES

TABLE S6 (EXCEL File) Comparison of EPTO (Ephemeroptera, Plecoptera, Trichoptera and Odonata) taxa detected at five sampling sites in the Tua River (Douro Basin, NE Portugal), using either morphological identification or metabarcoding. Comparisons are made separately for each of three extraction methods (BEAD, TISSUE and SOIL), and separately for each of the three replicates of the TISSUE extraction method.

MOLECULAR ECOLOGY
RESOURCES

TABLE S7 Summary statistics of GAMM models relating the Jaccard's distance between EPTO community compositions estimated from morphological and metabarcoding data, in relation to subsampling day and DNA extraction methods. Extractions were performed from the ethanol used to preserve five unprocessed freshwater macroinvertebrate bulk samples and subsampled on days 1, 2, 3, 5, 7 and 14 after field sampling, using three DNA extraction methods (BEAD, TISSUE and SOIL). For each model, we provide the parameter estimates, standard errors (SE) and statistical significance of parametric terms, and the effective degrees of freedom (edf) and approximate significance of smooth terms. Different models were built considering alternative criteria for dealing with rare species (details in the main text).

	**Jaccard Distance (Family)**				**Jaccard Distance (Species)**			
**Dataset using the raw data**								
Parametric coefficients	*Estimate*	*SE*	*t-value*	*P*	*Estimate*	*SE*	*t-value*	*P*
Intersect	0.630	0.058	10.821	$<2.0\times10^{-16}$***	0.744	0.052	14.223	$<2.0\times10^{-16}$***
TISSUE	0.048	0.025	1.966	0.050*	0.045	0.021	2.103	0.036*
SOIL	0.079	0.026	3.083	0.002**	0.068	0.022	3.063	0.002**
log(Reads)	-0.024	0.005	-4.590	5.9×10^{-06}***	-0.026	0.004	-5.812	1.2×10^{-08}***
Smooth terms	*edf*		*F-value*	*P*	*edf*		*F-value*	*P*
s(day):BEAD	1		3.818	0.051 *ns*	1		4.703	0.031*
s(day):TISSUE	2.064		17.703	3.8×10^{-08}***	2.669		17.905	9.6×10^{-09}***
s(day):SOIL	2.134		14.566	3.8×10^{-07}***	1.543		15.682	7.7×10^{-05}***
**Dataset excluding singletons from each sample**								
Parametric coefficients	*Estimate*	*SE*	*t-value*	*P*	*Estimate*	*SE*	*t-value*	*P*
Intersect	0.788	0.058	13.619	$<2.0\times10^{-16}$***	0.866	0.050	17.202	$<2.0\times10^{-16}$***
TISSUE	0.031	0.022	1.402	0.162 *ns*	0.032	0.021	1.552	0.121 *ns*
SOIL	0.064	0.023	2.722	0.007**	0.056	0.021	2.639	0.009**
log(Reads)	-0.039	0.005	-7.707	9.9×10^{-14}***	-0.038	0.004	-8.885	$<2.0\times10^{-16}$***
Smooth terms	*edf*		*F-value*	*P*	*edf*		*F-value*	*P*
s(day):BEAD	1		7.108	0.008**	1		13.120	3.3×10^{-04}***
s(day):TISSUE	2.140		22.672	2.4×10^{-10}***	2.325		21.920	3.2×10^{-10}***
s(day):SOIL	1.859		19.829	1.0×10^{-06}***	1.586		26.860	8.9×10^{-08}***
**Dataset excluding from each sample the taxa with a percentage of read counts <0.01%**								
Parametric coefficients	*Estimate*	*SE*	*t-value*	*P*	*Estimate*	*SE*	*t-value*	*P*
Intersect	0.610	0.058	10.418	$<2.0\times10^{-16}$***	0.731	0.052	14.123	$<2.0\times10^{-16}$***
TISSUE	0.034	0.021	1.596	0.111 *ns*	0.031	0.019	1.622	0.106 *ns*
SOIL	0.067	0.022	2.987	0.003**	0.060	0.020	2.992	0.003**
log(Reads)	-0.019	0.005	-3.640	3.1×10^{-04}***	-0.022	0.004	-5.200	3.2×10^{-07}***
Smooth terms	*edf*		*F-value*	*P*	*edf*		*F-value*	*P*
s(day):BEAD	1		7.411	0.007**	1		13.550	2.6×10^{-04}***
s(day):TISSUE	2.205		21.612	5.5×10^{-10}***	2.455		26.190	3.3×10^{-12}***
s(day):SOIL	2.032		16.302	1.2×10^{-07}***	1.381		31.240	1.5×10^{-08}***

****P*<0.001, ***P*<0.01, **P*<0.05, *ns P*>0.05.

TABLE S7 (cont.)

	Jaccard Distance (Family)				Jaccard Distance (Species)			
**Dataset excluding from each sample the taxa with a percentage of read counts <0.03%**								
Parametric coefficients	*Estimate*	*SE*	*t-value*	*P*	*Estimate*	*SE*	*t-value*	*P*
Intersect	0.586	0.056	10.374	$<2.0\times10^{-16}$***	0.704	0.050	14.11	$<2.0\times10^{-16}$***
TISSUE	0.020	0.024	0.856	0.392 *ns*	0.017	0.021	0.818	0.414 *ns*
SOIL	0.052	0.025	2.124	0.034*	0.045	0.021	2.097	0.036*
log(Reads)	-0.011	0.005	-2.130	0.034*	-0.014	0.004	-3.480	5.6×10^{-04}***
Smooth terms	*edf*		*F-value*	*P*	*edf*		*F-value*	*P*
s(day):BEAD	1		7.405	0.007**	1		11.660	7.0×10^{-04}***
s(day):TISSUE	2.349		22.065	3.1×10^{-10}***	2.403		21.010	6.3×10^{-10}***
s(day):SOIL	1.873		15.080	2.4×10^{-05}***	1.585		20.600	3.7×10^{-06}***
**Dataset excluding from each sample the taxa with a percentage of read counts <0.05%**								
Parametric coefficients	*Estimate*	*SE*	*t-value*	*P*	*Estimate*	*SE*	*t-value*	*P*
Intersect	0.593	0.054	11.069	$<2.0\times10^{-16}$***	0.692	0.049	14.262	$<2.0\times10^{-16}$***
TISSUE	0.008	0.025	0.337	0.736 *ns*	0.012	0.022	0.548	0.584 *ns*
SOIL	0.048	0.027	1.800	0.073 *ns*	0.048	0.023	2.084	0.038*
log(Reads)	-0.008	0.005	-1.633	0.103 *ns*	-0.010	0.004	-2.538	0.012*
Smooth terms	*edf*		*F-value*	*P*	*edf*		*F-value*	*P*
s(day):BEAD	1.507		2.033	0.092 *ns*	1		6.024	0.014*
s(day):TISSUE	2.420		22.519	1.7×10^{-10}***	2.447		20.517	1.0×10^{-09}***
s(day):SOIL	1.910		16.592	2.4×10^{-06}***	1.106		27.333	2.0×10^{-07}***

****P*<0.001, ***P*<0.01, **P*<0.05, *ns P*>0.05.

MOLECULAR ECOLOGY RESOURCES

TABLE S8 Summary statistics of GAMM models relating the percentage matching between EPTO community compositions estimated from morphological and metabarcoding data, in relation to subsampling day and DNA extraction methods. Extractions were performed from the ethanol used to preserve five unprocessed freshwater macroinvertebrate bulk samples and subsampled on days 1, 2, 3, 5, 7 and 14 after field sampling, using three DNA extraction methods (BEAD, TISSUE and SOIL). For each model, we provide the parameter estimates, standard errors (SE) and statistical significance of parametric terms, and the effective degrees of freedom (edf) and approximate significance of smooth terms. Different models were built considering alternative criteria for dealing with rare species (see the main text for details).

	**Percentage Matching (Family)**				**Percentage Matching (Species)**			
**Dataset using the raw data**								
Parametric coefficients	*Estimate*	*SE*	*t-value*	*P*	*Estimate*	*SE*	*t-value*	*P*
Intersect	0.464	0.067	6.963	1.3×10^{-11}***	0.202	0.059	3.443	0.001***
TISSUE	-0.065	0.039	-1.680	0.094 *ns*	-0.067	0.033	-2.008	0.045*
SOIL	-0.114	0.040	-2.866	0.004**	-0.111	0.034	-3.285	0.001**
log(Reads)	0.031	0.006	5.156	3.9×10^{-07}***	0.052	0.006	9.325	$<2.0\times10^{-16}$***
Smooth terms	*edf*		*F-value*	*P*	*edf*		*F-value*	*P*
s(day):BEAD	1		5.076	0.025*	1.334		5.404	0.009**
s(day):TISSUE	2.322		22.816	7.7×10^{-11}***	2.550		27.554	7.3×10^{-13}***
s(day):SOIL	2.260		23.147	7.4×10^{-11}***	1.777		22.162	5.0×10^{-07}***
**Dataset excluding singletons from each sample**								
Parametric coefficients	*Estimate*	*SE*	*t-value*	*P*	*Estimate*	*SE*	*t-value*	*P*
Intersect	0.365	0.068	5.352	1.5×10^{-07}***	0.081	0.056	1.448	0.148 *ns*
TISSUE	-0.051	0.032	-1.594	0.112 *ns*	-0.043	0.028	-1.516	0.130 *ns*
SOIL	-0.094	0.033	-2.868	0.004**	-0.084	0.029	-2.894	0.004**
log(Reads)	0.038	0.006	6.412	4.0×10^{-10}***	0.059	0.005	11.090	$<2.0\times10^{-16}$***
Smooth terms	*edf*		*F-value*	*P*	*edf*		*F-value*	*P*
s(day):BEAD	1		3.886	0.049*	1		16.390	6.1×10^{-05}***
s(day):TISSUE	2.258		24.489	1.9×10^{-11}***	2.343		30.110	7.0×10^{-14}***
s(day):SOIL	2.120		21.701	6.9×10^{-10}***	1.926		26.770	1.4×10^{-09}***

****P*<0.001, ***P*<0.01, **P*<0.05, *ns P*>0.05.

MOLECULAR ECOLOGY RESOURCES

TABLE S8 (cont.)

	Percentage Matching (Family)				Percentage Matching (Species)			
**Dataset excluding from each sample the taxa with a percentage of read counts <0.01%**								
Parametric coefficients	*Estimate*	*SE*	*t-value*	*P*	*Estimate*	*SE*	*t-value*	*P*
Intersect	0.520	0.068	7.685	1.2×10^{-13}***	0.303	0.057	5.277	2.1×10^{-07}***
TISSUE	-0.052	0.032	-1.634	0.103 *ns*	-0.048	0.026	-1.839	0.067 *ns*
SOIL	-0.098	0.033	-2.997	0.003**	-0.094	0.027	-3.514	4.9×10^{-04}***
log(Reads)	0.020	0.006	3.403	7.3×10^{-04}***	0.033	0.005	6.399	4.3×10^{-10}***
Smooth terms	*edf*		*F-value*	*P*	*edf*		*F-value*	*P*
s(day):BEAD	1		5.565	0.019*	1		17.270	3.9×10^{-05}***
s(day):TISSUE	2.315		26.032	2.9×10^{-12}***	2.458		34.130	7.7×10^{-16}***
s(day):SOIL	2.173		23.066	1.2×10^{-10}***	1.822		28.600	4.0×10^{-09}***
**Dataset excluding from each sample the taxa with a percentage of read counts <0.03%**								
Parametric coefficients	*Estimate*	*SE*	*t-value*	*P*	*Estimate*	*SE*	*t-value*	*P*
Intersect	0.494	0.069	7.193	3.0×10^{-12}***	0.321	0.056	5.747	1.8×10^{-08}***
TISSUE	-0.026	0.029	-0.892	0.373 *ns*	-0.019	0.026	-0.714	0.476 *ns*
SOIL	-0.073	0.030	-2.408	0.016*	-0.065	0.027	-2.365	0.019*
log(Reads)	0.017	0.006	2.970	0.003**	0.023	0.005	4.419	1.3×10^{-05}***
Smooth terms	*edf*		*F-value*	*P*	*edf*		*F-value*	*P*
s(day):BEAD	1		3.833	0.051 *ns*	1		10.010	0.002**
s(day):TISSUE	2.262		26.708	3.1×10^{-12}***	2.440		27.890	5.2×10^{-13}***
s(day):SOIL	1.978		23.914	1.4×10^{-09}***	1.941		20.170	1.5×10^{-07}***
**Dataset excluding from each sample the taxa with a percentage of read counts <0.05%**								
Parametric coefficients	*Estimate*	*SE*	*t-value*	*P*	*Estimate*	*SE*	*t-value*	*P*
Intersect	0.498	0.068	7.362	1.0×10^{-12}***	0.345	0.054	6.435	3.5×10^{-10}***
TISSUE	-0.013	0.030	-0.428	0.668 *ns*	-0.010	0.028	-0.361	0.719 *ns*
SOIL	-0.072	0.031	-2.338	0.020*	-0.063	0.030	-2.136	0.033*
log(Reads)	0.013	0.006	2.295	0.022*	0.015	0.005	3.142	0.002**
Smooth terms	*edf*		*F-value*	*P*	*edf*		*F-value*	*P*
s(day):BEAD	1.245		1.075	0.247 *ns*	1		4.928	0.027*
s(day):TISSUE	2.265		21.171	8.6×10^{-10}***	2.488		26.967	1.0×10^{-12}***
s(day):SOIL	1.595		25.914	1.1×10^{-07}***	1.748		22.602	3.7×10^{-07}***

****P*<0.001, ***P*<0.01, **P*<0.05, *ns P*>0.05.

MOLECULAR ECOLOGY RESOURCES

Supplementary Figures

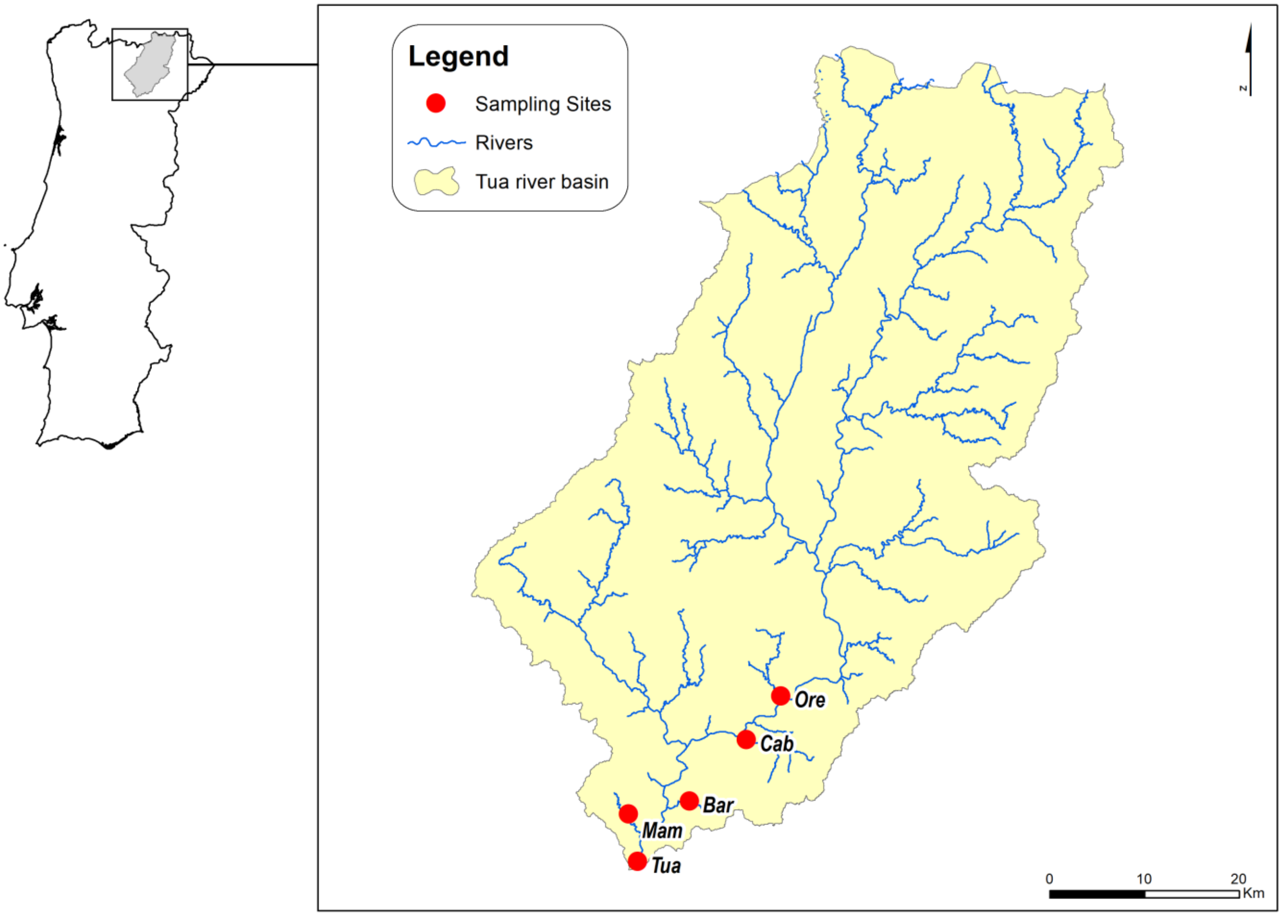

FIGURE S1 Map of the study area showing the location of the five sites sampled in 2015 for benthic freshwater macroinvertebrates in the Tua watershed (Douro Basin, NE Portugal)
